# A simple and efficient system for evaluating plant genome editing efficiency and its application in optimizing the ISAam1 TnpB nuclease

**DOI:** 10.3389/fpls.2025.1620874

**Published:** 2025-09-01

**Authors:** Xingyu Cao, Shasha Bai, Jun Li, Yongwei Sun

**Affiliations:** Key Laboratory of Herbage and Endemic Crop Biology, Ministry of Education, Inner Mongolia University, Hohhot, Inner Mongolia, China

**Keywords:** genome editing, *Agrobacterium rhizogenes*, plants, ISAam1, TnpB

## Abstract

Genome editing technology has revolutionized plant genetic breeding. However, Significant variability in editing activity has been observed across different genome editing systems and target sites, highlighting the importance of developing efficient evaluation systems for assessing genome editing efficiency in plants. In this study, we developed a simple, rapid, and efficient system based on hairy root transformation to evaluate somatic genome editing efficiency in plants. This system is easy to implement, does not require sterile conditions, and enables visual identification of transgenic hairy roots within two weeks. We first validated the system using the CRISPR/Cas9 genome editing platform, confirming its effectiveness. Subsequently, we applied this system to assess the somatic editing activity of the recently identified ISAam1 TnpB nuclease, which show considerable promise for plant genome editing applications. Furthermore, through protein engineering, we identified two variants, ISAam1(N3Y) and ISAam1(T296R), which exhibited a 5.1-fold and 4.4-fold enhancement in somatic editing efficiency, respectively. These findings demonstrate that the developed method provides an effective tool for optimizing genome editing system and screening potential target sites in plant genomes.

## Introduction

CRISPR/Cas technology has revolutionized the field of life sciences. However, in contrast to the extensive research conducted in mammalian cells, the development and application of novel CRISPR/Cas systems in plants have lagged behind their counterparts. Most CRISPR systems have been initially developed for genome editing in human cells and subsequently adapted for plant applications (Zhong et al., 2023). However, genome editing in plants presents distinct challenges compared to mammalian systems. Many editing systems that demonstrate high efficiency in mammalian cells either fail to work or exhibit marked reductions in editing efficiency when applied in plants (Zhong et al., 2023). As a result, developing a system that allows for rapid evaluation of genome editing efficiency in plants is critically important. In many cases, the *in vitro* cleavage assay in protoplasts offers a convenient method for assessing genome editing efficiency (Li et al., 2013, 2017b; Molla et al., 2021; Pan et al., 2021; Panda et al., 2024; Perroud et al., 2023; Syombua et al., 2021; Xiong et al., 2023; Zhang et al., 2022). However, the routine use of protoplasts in laboratory settings faces several limitations, including the complexity of the isolation process, low viability of isolated protoplasts, and suboptimal transfection efficiency. Moreover, protoplast-based assays typically rely on transient expression systems, which may not accurately reflect the true genome editing efficiency observed in stably transformed plants.

Hairy root transformation mediated by *Agrobacterium rhizogenes* offers a more efficient, rapid, and straightforward alternative to *Agrobacterium tumefaciens*-mediated transformation. Following infection, the characteristic “hairy root syndrome” is induced, resulting in the formation of chimeric composite plants with transgenic roots and non-transgenic shoots within just a few weeks (Gutierrez-Valdes et al., 2020; Kereszt et al., 2007; Kong et al., 2023). It has also been widely applied in the field of plant genome editing, particularly in the efficient screening of genome editing sites (Bai et al., 2024; Cao et al., 2023). However, for many studies, *Agrobacterium*-mediated genome editing still requires operation under sterile conditions to obtain transgenic hairy roots, which is time-consuming and labor-intensive (Zhu et al., 2024). This poses significant challenges for large-scale target screening or system optimization experiments of genome editing nucleases. Recent studies have reported that soybean transgenic hairy roots can be rapidly obtained through a one-step method that does not require aseptic conditions (Fan et al., 2020). However, the infection process and screening for transgenic positive hairy roots still require significant effort.

In this study, we developed a simple and rapid system for evaluating somatic genome editing activity in plants, capable of producing transgenic hairy roots within two weeks. This system is easy to operate, does not require sterile conditions, and enables clear identification of transgenic hairy roots without the need for specialized instruments or equipment. Furthermore, we applied this method to assess the somatic genome editing efficiency of the small nuclease ISAam1 TnpB in plants. Through protein engineering, we identified two variants, ISAam1(N3Y) and ISAam1(T296R), which exhibited significantly enhanced somatic editing efficiency. Collectively, this system provides a practical and efficient platform for evaluating and optimizing somatic genome editing tools in plants.

## Results

### Development of a system for the rapid generation of transgenic hairy roots

**Figure 1:**
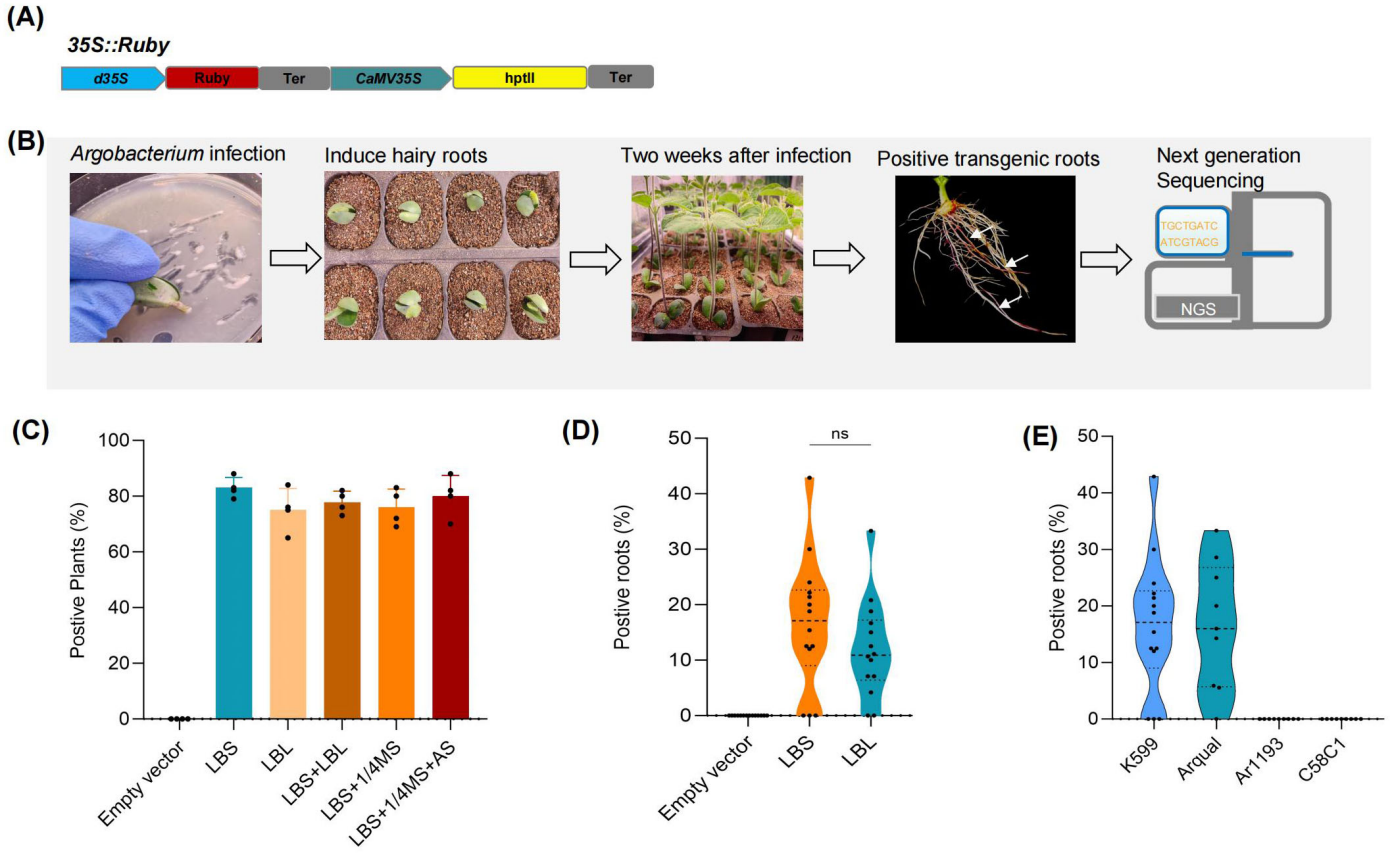
Development of an efficient method for the generation of transgenic hairy roots. **(A)** Schematic representation of the 35S: ruby vector for the expression of Ruby. **(B)** Workflow of soybean hairy root transformation protocol. **(C)** Impact of different infection methods on transformation efficiency. Each point represents a biological replicate from an independent experiment, in which no fewer than 30 plants were inoculated. **(D)** Number of positive roots per independent plant across various infection methods. **(E)** Effect of different Agrobacterium rhizogenes strains on the transformation efficiency of transgenic hairy roots. Data were analyzed using one-way ANOVA, assuming Gaussian distribution of residuals and equal standard deviations among groups; ns, P>0.05; Data are presented as mean values ± SD.

In this study, we first established a simple and efficient hairy root transformation system using soybean as the model organism. This system involved slant cut of the hypocotyl of soybeans germinated for 5–7 days and directly infecting them with *Agrobacterium rhizogenes* harboring *35S:Ruby* vectors capable of expressing the *Ruby* gene (He et al., 2020), which is a synthetic reporter gene system to track gene expression and successful plant transformations without needing special equipment (**Figure 1A**), followed by cultivation in moist vermiculite. After two weeks, we were able to visually select transgenic soybean roots (**Figure 1B**).

For the infection protocol, the slant cut of the hypocotyl was either scraped onto Luria-Bertani solid medium containing K599 *Agrobacterium rhizogenes* (LBS), directly planted into vermiculite and watered with K599 liquid medium (LBL), or watered with resuspended *Agrobacterium rhizogenes* in 1/4 Murashige and Skoog liquid medium (1/4 MS), or watered with 1/4 MS supplemented with 100 μmol of Acetosyringone (1/4 MS + AS), or subjected to a combination of these methods (LBS+LBL; LBS+1/4MS; LBS+1/4MS+AS). Our study demonstrated that all infection protocols resulted in a high rate of successful transformation, with 80% of the infected plants exhibiting transformed roots (**Figure 1C**). Moreover, within each infected plant, 10% of the roots were successfully transformed (**Figure 1D**). Given that different *Agrobacterium rhizogenes* strains typically show significant variations in infection efficiency across different plant species, we applied the LBS infection method to three *Agrobacterium rhizogenes* strains: Ar1193, Arqual, and C58C1. The results indicated that the infection efficiency of these three strains was lower than that of K599 in soybean (**Figure 1E**).

**Figure 2:**
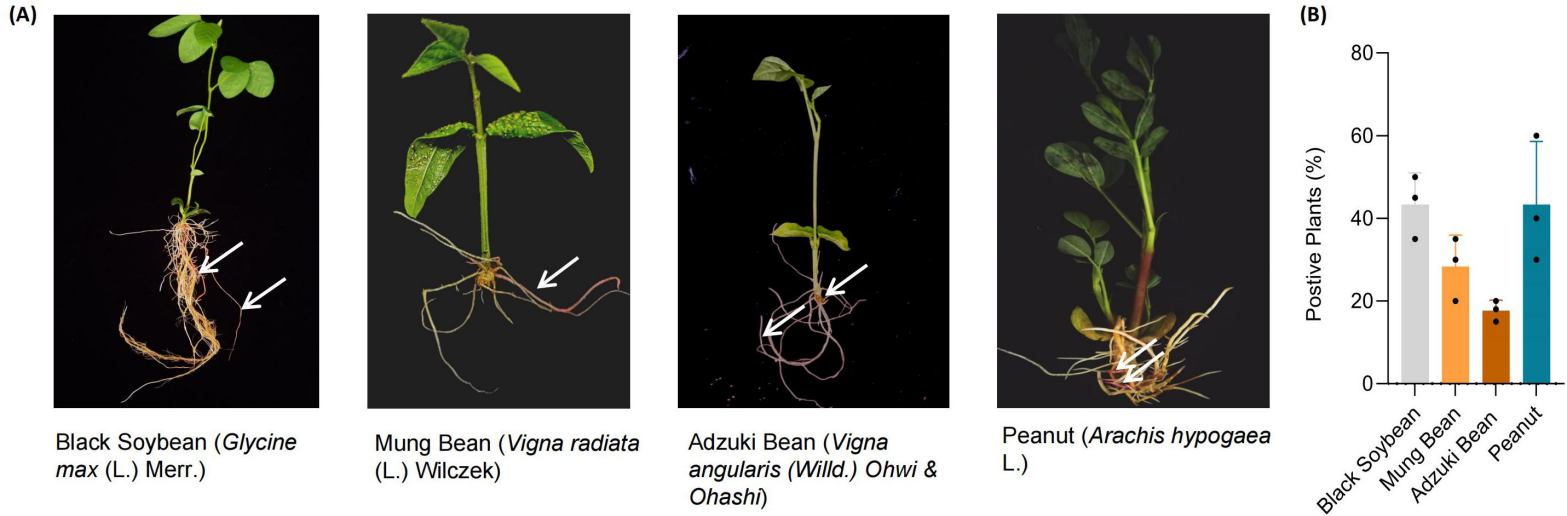
Hairy root transformation of black soybean, mung bean, adzuki bean, and peanut mediated by Agrobacterium rhizogenes. **(A)** Positive transgenic hairy roots in black soybean, mung bean, adzuki bean and peanut, respectively. The white arrows indicate the transgenic hairy roots, which exhibit a red coloration due to the stable expression of the Ruby gene. **(B)** Agrobacterium rhizogenes K599 mediated hairy root transformation efficiency in black soybean, mung bean, adzuki bean and peanut. Each point represents a biological replicate from an independent experiment, in which no fewer than 20 plants were inoculated.

Studies have shown that *Agrobacterium rhizogenes* can infect most dicots, a small number of monocots, and gymnosperms. To date, hairy roots have been developed and exploited in more than 400 plant species across 50 angiosperm families and 150 genera, with the majority concentrated in the *Apiaceae, Asteraceae, Brassicaceae, Caryophyllaceae, Convolvulaceae, Fabaceae, Polygonaceae, and Solanaceae* families (Phuong et al., 2023; Rogowska and Szakiel, 2021; Banerjee et al., 2012; Wan et al., 2023; Zhu et al., 2024). To assess whether our established method is applicable across a broader range of plant species, we selected several plant species for verification, including peanut (*Arachis hypogaea* L.), adzuki bean (*Vigna angularis* (Willd.) Ohwi & Ohashi), mung bean (*Vigna radiata* (L.) Wilczek), and black soybean (*Glycine max* (L.) Merr.). These species were subjected to transformation using *Agrobacterium rhizogenes* strain K599 for hairy root induction. The results demonstrated that the method was similarly effective across all tested species, with transformation efficiencies of 43.3% in black soybean, 28.3% in mung bean, 17.7% in adzuki bean, and 43.3% in peanut (**Figure 2**).

### Evaluation of CRISPR/Cas9-mediated somatic genome editing efficiency using *Agrobacterium rhizogenes*-induced hairy root systems

**Figure 3:**
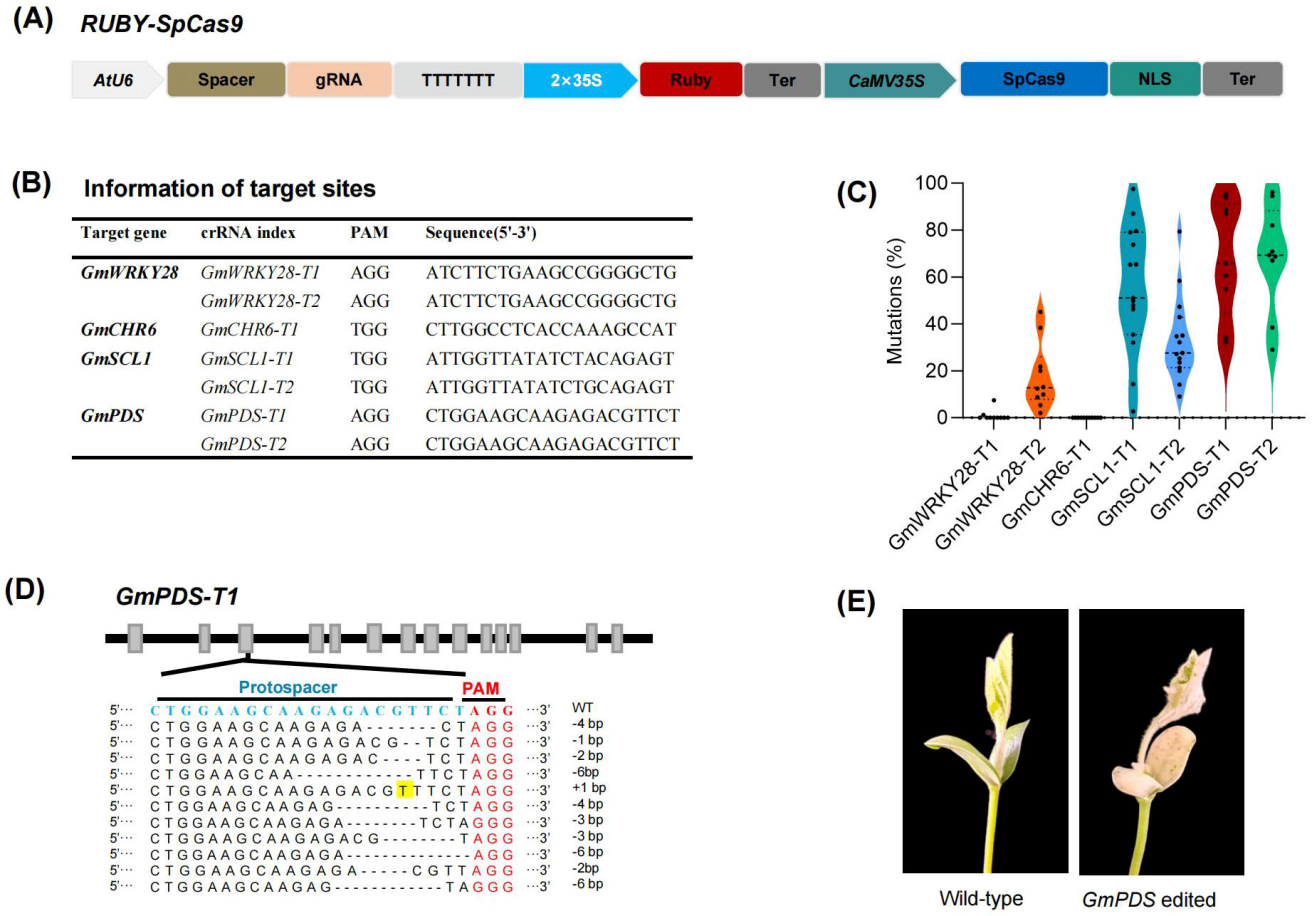
Development of a system for the rapid evaluation of genome editing efficiency in plants. **(A)** Schematic representation of the RUBY-SpCas9 vector for the co-expression of Ruby, SpCas9, and sgRNA. **(B)** Information of seven target sites. **(C)** Genome editing efficiency of the CRISPR/Cas9 system targeting seven loci in transgenic hairy roots. Each point represents the genome editing efficiency of an independent transgenic hairy root (n> 8). **(D)** Mutations type of CRISPR/Cas9-mediated genome editing in soybean hair roots. The PAM sequences and spacers are highlighted in red and blue, respectively. Dashes represent deletions, while insertions are shaded in yellow. **(E)** Phenotypes of GmPDS gene edited mutants.

Next, we sought to verify whether our method could rapidly evaluate the efficiency of somatic genome editing. We constructed a CRISPR/Cas9 system into the *35S:Ruby* vectors targeting endogenous loci within the *GmWRKY28*, *GmCHR6*, *GmPDS1, GmPDS2* and *GmSCL1* gene (**Figures 3A, B**). The results from next-generation sequencing (NGS) demonstrated that 5 out of 7 targets showed high somatic editing efficiency (**Figure 3C**). Notably, although the target sequences of GmWRKY28-T1 and GmWRKY28-T2 are identical, the somatic genome editing efficiency varied significantly between the homologous genes. No somatic genome editing activity was detected at GmWRKY28-T1, whereas at GmWRKY28-T2, the somatic editing efficiency reached as high as 45.1%, with an average of 13.1% somatic genome editing efficiency (**Figure 3C**). This highlights the importance of screening for highly efficient genome editing sites before initiating stable transformation. Analysis of the genome editing types in individual transgenic hairy roots revealed that the genome editing observed was predominantly chimeric (**Figure 3D**). This may be attributed to the fact that these transgenic roots were developed without undergoing traditional tissue culture, antibiotic selection, and regeneration processes. Consequently, this method is particularly well-suited for evaluating genome editing efficiency because each root represents a complex assembly of numerous transgenic cells, thus providing a more accurate reflection of genome editing characteristics.

To further evaluate the feasibility and applicability of the method established in this study, the CRISPR/Cas9 construct, designed to target the *GmPDS1* and *GmPDS2* genes and in which the *RUBY* gene was replaced by the *bar* gene as a selectable marker, was subsequently introduced into soybean via *Agrobacterium tumefaciens*-mediated transformation. Following tissue culture and plant regeneration, ten independent stable transgenic lines were successfully obtained. Next generation sequencing (NGS) of the editing sites revealed that eight of these lines carried mutations at the expected loci. Phenotypic characterization showed that several edited plants exhibited mild chlorotic phenotypes, whereas distinct albino phenotypes were observed in T_1_ generation plants (**Figure 3E**). These results further demonstrate the feasibility and potential applications of the method established in this study.

### Characterization and engineering of ISAam1 TnpB-mediated somatic genome editing in soybean hairy roots

**Figure 4:**
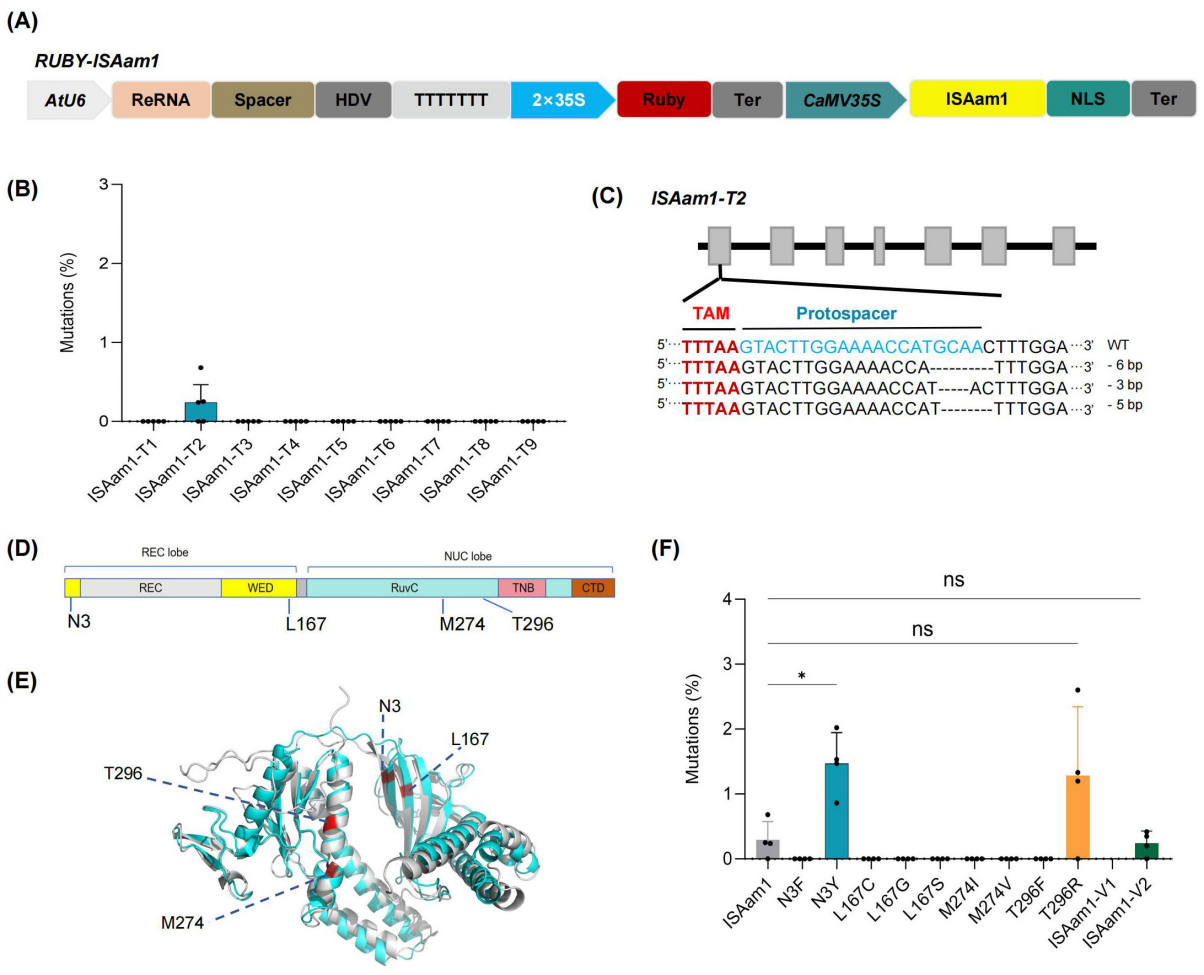
Engineering ISAam1 for efficient genome editing in plants. **(A)** Schematic representation of the RUBY-ISAaml vector for the co-expression of Ruby, ISAam 1, and ReRNA. **(B)** Genome editing efficiency of the ISAaml targeting ten loci in transgenic hairy roots. Each point represents a biological replicate from an independent experiment (n > 3). **(C)** Mutations type of ISAam1-mediated genome editing in soybean hair roots. The TAM sequences and spacers are highlighted in red and blue, respectively. Dashes represent deletions. **(D)** Domain organization of ISAam1. REC, recognition domain; WED, wedge; RuvC, RuvC endonuclease domain; TNB, target nucleic acid-binding; CTD, C-terminal domain. **(E)** Identification of sites that enhance genome editing efficiency of ISAam. The protein structures of ISDra2 (gray) and ISAaml (cyan) are shown, with red highlights indicating the sites in ISDra2 that improve genome editing efficiency and their corresponding sites in ISAam1. **(F)** Editing efficiency of ISAam variants. Each point represents the genome editing efficiency of an independent transgenic hairy root (n > 3). Data were analyzed using one-way ANOVA. Statistical significance is denoted as follows: ns, P > 0.05; *, P < 0.05; Data are presented as mean values ± SD.

The widely used CRISPR/Cas system is thought to have evolved from IS200/IS605 transposons. TnpB proteins, encoded by one type of IS200/IS605 transposon, are considered to be the evolutionary ancestors of Cas12 nucleases, which have been engineered to function as RNA-guided DNA endonucleases for genome editing in bacteria and human cells, and have recently been reported in plants, including *Arabidopsis*, rice, and medicinal plants. However, the efficiency remains relatively low, with significant variations observed across different targets, indicating that there is still considerable room for improvement (Karmakar et al., 2024; Lv et al., 2024; Weiss et al., 2025; Zhang et al., 2024). ISAam1 TnpB is a member of the TnpB protein family. To date, ISAam1-mediated genome editing has been evaluated exclusively in rice and *Arabidopsis thaliana*. Notably, the editing efficiency observed in rice protoplasts ranged from 2.36% to 4.65%. In *Arabidopsis thaliana*, among the 20 target sites tested, only 7 exhibited detectable editing activity, with efficiencies ranging from 0% to 0.3% (Lv et al., 2024; Weiss et al., 2025; Zhang et al., 2024). To evaluate the feasibility of the method developed in this study for engineering novel nucleases, we cloned the rice codons optimized *ISAam1* gene into the *35S:Ruby* vector and constructed nine target-specific editing constructs for the *GmBADH1*, *GmSweet15*, *GmFAD2-1A*, and *GmCCD4* genes. These constructs were then introduced into soybean hairy roots for further analysis (**Figure 4A**). The results indicated that, among the nine selected targets, only one target (ISAam1-T2) exhibited somatic genome editing, with an average editing efficiency of just 0.29% (**Figures 4B, C**). These findings suggest that there is still considerable room for improvement before these nucleases can be effectively applied in soybean.

Recent studies have demonstrated that engineered optimization of ISDra2 TnpB can substantially enhance its genome editing activity in both animal and plant systems. Moreover, the amino acid residues associated with improved editing efficiency have been found to be highly conserved across various TnpB homologs (Thornton et al., 2025). Guided by protein sequence alignment and structural predictions generated by *AlphaFold2*, we found that, ISAam1 TnpB and ISDra2 TnpB exhibit a high degree of structural similarity, with a root mean square deviation (RMSD) values of 1.68. Based on these findings, we identified four potentially critical sites in ISAam1 that may influence genome editing efficiency (**Figures 4D, E**). Based on these insights, we rationally engineered ten variants (N3F, N3Y, L167C, L167G, L167S, M274I, M274V, T296F, T296R, and a combined variant ISAam1-V1 [N3Y/L167G/M274I/T296R]). Genome editing constructs targeting the ISAam1-T2 locus were generated for each variant. Using the rapid hairy root transformation system developed in this study, we screened transgenic hairy roots and performed NGS of the target site. The results revealed that variants N3Y and T296R significantly enhanced somatic genome editing efficiency, achieving 1.47% (5.1-fold increase) and 1.28% (4.4-fold increase), respectively. However, the combination of these two mutations in ISAam1-V2 led to a reduction in somatic editing efficiency in ISAam1-T2, rather than a further enhancement (**Figure 4F**).

## Discussion

The assessment of genome editing efficiency, particularly in the context of applying and engineering novel editing systems in plants, often necessitates the construction of numerous expression vectors for validation. Consequently, the development of a streamlined, efficient, and robust method for such evaluations is critically important. In comparison to the transient protoplast transformation method, *Agrobacterium rhizogenes*-mediated transformation presents clear advantages. This approach eliminates the requirement for large-scale purification of high-copy plasmid DNA and avoids the technically demanding process of protoplast isolation. It is operationally streamlined and, more importantly, achieves a high transformation efficiency. Notably, the genome editing efficiency achieved with this system was comparable to that observed in stable transgenic. For the constructs targeting the *GmPDS1* and *GmPDS2* genes, all nine tested independent transgenic hairy roots exhibited detectable genome editing activity, while eight out of ten stable transgenic soybean lines harboring the same construct also displayed editing activity. Previously, Cao et al. (2023) developed a highly efficient and simple cut-dip-budding (CDB) system, which involves the inoculation of explants with *Agrobacterium rhizogenes* to induce the formation of transgenic roots. Transgenic plants are subsequently generated through adventitious rooting. This method successfully facilitated genetic transformation and genome editing in several plant species (Cao et al., 2023; Gu et al., 2025; Lu et al., 2024; Sun et al., 2025). However, for these plants, the process from infection to the generation of transgenic hairy roots still requires a relatively long period. Additionally, their genomic information remains relatively underexplored, making them less suitable for rapid evaluation of genome editing systems. In this study, we focused on soybean as a model organism with well-defined genomic information and developed a rapid method for generating stable transgenic roots. This method not only eliminates the need for sterile procedures, but also allows for the direct visual identification of transgenic hairy roots. More importantly, it enables the generation of transgenic hairy roots within two weeks. Undoubtedly, this approach will significantly accelerate the development and application of plant genome editing systems.

ISAam1 is distinguished by its recognition of a specific target adjacent motif (TAM), TTTAA, which sets it apart from other TnpB orthologs, such as ISDra2, ISYmu1, and ISDge10. These orthologs recognize the motifs TTGAT, TTGAT, and TTAT, respectively. In addition to its unique TAM specificity, ISAam1TnpB is characterized by a notably compact structure, comprising only 369 amino acids. In contrast, ISDra2, ISYmu1, and ISDge10-TnpB contain 408, 381, and 391 amino acids, respectively (Xiang et al., 2024; Zhang et al., 2024). Due to its compact size, ISAam1 holds great potential for broader applications, particularly in virus-mediated genome editing (Hu et al., 2025; Liu and Zhang, 2020; Tuncel et al., 2025). In this study, we demonstrated that ISAam1 possesses somatic genome editing activity in soybean. Furthermore, structural prediction using *AlphaFold2* enabled the identification of putative residues that may enhance its somatic editing activity. By employing the evaluation system established in this study, we efficiently identified two ISAam1 variants with significantly improved somatic genome editing efficiency, which not only demonstrates the applicability of this system but also provides a solid foundation for its further use in plant genome engineering. However, it is important to transparently acknowledge the current limitations and challenges associated with applying the ISAam1 TnpB system, even with the optimized variants, particularly in the context of generating heritably edited plants. To address the current limitation of mutation frequency, rational protein engineering guided by structural insights may be pursued to further enhance the editing efficiency of ISAam1 TnpB. Future work will therefore focus on systematic site-directed mutagenesis or directed evolution targeting these predicted functional domains, such as the catalytic RuvC-like nuclease domain, the TAM recognition interface, and regions involved in guide RNA binding or conformational changes. It is anticipated that such comprehensive engineering approaches will significantly enhance the genome editing efficiency of ISAam1 TnpB in plants.

## Methods

### Vector construction

The vectors used in this study were constructed based on the backbone of *35S:Ruby* (Addgene#160908) from Yubing He’s lab. The DNA sequences for the *ISAam1 TnpB* gene were optimized for rice codons and synthesized by Sangon Biotech (Shanghai, China). Different variants of the *ISAam1* gene were generated through overlapping PCR to introduce specific mutations. For testing editing activity in soybean hairy roots, target sites were selected based on the reference sequence of the relevant gene and the requirements of the TAM sequence. Once a target site was selected, forward and reverse oligonucleotides were designed and synthesized. These oligonucleotides were then used to generate the reRNA scaffold via overlapping PCR. The reRNAs and *ISAam1* gene were driven by *AtU6* and *CaMV35S* promoter, respectively. All DNA fragments were assembled into the linearized *35S:Ruby* backbone using Seamless Cloning (TransGen Biotech). The full-length sequences and maps of *RUBY-SpCas9* and *RUBY-ISAam1* are provided in the Supporting Information.

### Hairy roots transformation

The constructed plasmids were introduced into various *Agrobacterium rhizogenes* competent cells using the calcium chloride (CaCl_2_)-mediated transformation method. Single colonies were selected, cultured in liquid medium with shaking, and subsequently aliquoted into 2.0 mL centrifuge tubes for storage and further use. *Agrobacterium rhizogenes* used for plant infection were prepared using the following methods: (1) A tube of *Agrobacterium rhizogenes* glycerol stock was spread onto LB solid medium containing the appropriate antibiotics (LBS); (2) A 20μL aliquot of glycerol stock was inoculated into 5mL of LB liquid medium and incubated overnight at 28°C with shaking. The resulting culture was either used directly for plant infection (LBL) or centrifuged and resuspended in 1/4 MS liquid medium supplemented with 100μM acetosyringone (1/4 MS + AS). All preparations for infection were conducted one day prior to plant inoculation to ensure optimal bacterial activity and infection efficiency. Competent cells of *Agrobacterium rhizogenes* strains K599, Ar1193, Arqual, and C58C1, along with associated chemicals, were commercially procured from Coolaber Co., Ltd. (Beijing, China).

Soybean transgenic hairy roots were generated using a modified version of a previously reported protocol (Fan et al., 2020). Briefly, healthy seedlings at 5–7 days post-germination, with fully expanded true leaves, were selected for transformation. The primary root was removed using sterile scissors, leaving a 0.7–1 cm segment of the hypocotyl intact. The wounded surface was either scraped onto LB solid medium containing K599 *Agrobacterium rhizogenes* (LBS), directly planted into vermiculite and watered with K599 liquid medium (LBL), or watered with resuspended *Agrobacterium rhizogenes* in 1/4 Murashige and Skoog liquid medium (1/4 MS), or watered with 1/4 MS supplemented with 100 μmol of Acetosyringone (1/4 MS + AS), or subjected to a combination of these methods (LBS+LBL; LBS+1/4MS; LBS+1/4MS+AS). The inoculated plants were subsequently maintained under high-humidity conditions to promote root induction. Emergence of hairy roots was typically observed within two weeks post-inoculation.

The generation of transgenic hairy roots in black soybean, mung bean, adzuki bean, and peanut was conducted following protocols established for soybean, with appropriate modifications for each species. For black soybean, mung bean, and adzuki bean, seeds were germinated for approximately 7–10 days until the seedlings developed fully expanded true leaves. Seedlings with well-developed leaves were selected for transformation. The primary root was removed using sterile scissors, leaving a 0.5–0.8 cm segment of the hypocotyl intact. The wound site was thoroughly coated with *Agrobacterium rhizogenes* strain K599 harboring the *35S:Ruby* vector, and the seedlings were directly inserted into moist vermiculite. Following cultivation under these conditions for two weeks, transgenic hairy roots could be visually identified. For peanut, seeds were sown and grown for approximately 20 days to obtain robust seedlings. The aerial part of the seedling was excised and the cut surface was coated with *Agrobacterium rhizogenes* K599 harboring *35S:Ruby* vector. The treated shoots were then inserted into moist vermiculite. Transgenic hairy roots typically emerged after approximately one month. Throughout this period, vermiculite moisture was consistently maintained to ensure successful root induction.

### *Agrobacterium tumefaciens*-mediated stable transformation of soybean

To construct vectors targeting the *GmPDS1* and *GmPDS2* genes, the *RUBY* reporter gene in the *RUBY-SpCas9* vector was replaced with the *bar* gene to enable selection of transgenic positive plants. The resulting constructs were introduced into *Agrobacterium tumefaciens* strain EHA105 and subsequently transformed into Williams 82, following previously established protocols (Li et al., 2017a).

### Mutagenesis analysis

Mutation frequencies in soybean hairy roots were determined via amplicon-based deep sequencing, as described previously with slight modifications (Sun et al., 2024). Genomic DNA was extracted and used as a template for PCR amplification. The resulting amplicons were submitted to the Hi-TOM platform at the State Key Laboratory of Rice Biology and Breeding, China National Rice Research Institute, Chinese Academy of Agricultural Sciences (Hangzhou, China), for sequencing. A minimum of 5,000 reads per sample were obtained for mutation efficiency determination. The mutation frequency was calculated as the percentage of reads harboring insertions or deletions (indels) within the target site sequence and its flanking 20-bp regions on both sides. Mutation frequencies were calculated using data exported from the Hi-TOM platform and analyzed in Microsoft Excel.

Statistical analyses were conducted using GraphPad Prism version 8.0, and graphical outputs were refined and assembled using Adobe Photoshop and Adobe Illustrator.

### Protein structures alignments and analyzing

The ISAam1 and ISDra2 protein structures were predicted using Alphafold v2.2.0. Protein structure visualizations and diagrams were generated using PyMOL (DeLano, 2002).

## Data Availability

The original contributions presented in the study are included in the article/**Supplementary Material**. Further inquiries can be directed to the corresponding authors.

## Supplementary material

Supplementary Figure 1 Maps of RUBY-SpCas9 and RUBY-IsAam1.

Supplementary Table 1 Sequences of RUBY-SpCas9 and RUBY-IsAam1.

Supplementary Table 2 Amino acid sequence of ISAam1.

Supplementary Table 3 Primer sets used in this study.
