# Supplementary material for: A simple and efficient system for evaluating plant genome editing efficiency and its application in optimizing the ISAam1 TnpB nuclease
Source: Front Plant Sci. 2025 Sep 1;16:1620874. doi: 10.3389/fpls.2025.1620874 (PMC12433972; doi:10.3389/fpls.2025.1620874)
Supplement: Supplementary Figure 1 — Maps of RUBY-SpCas9 and RUBY-IsAam1. [file DataSheet1.docx]

**Supplementary information**

**
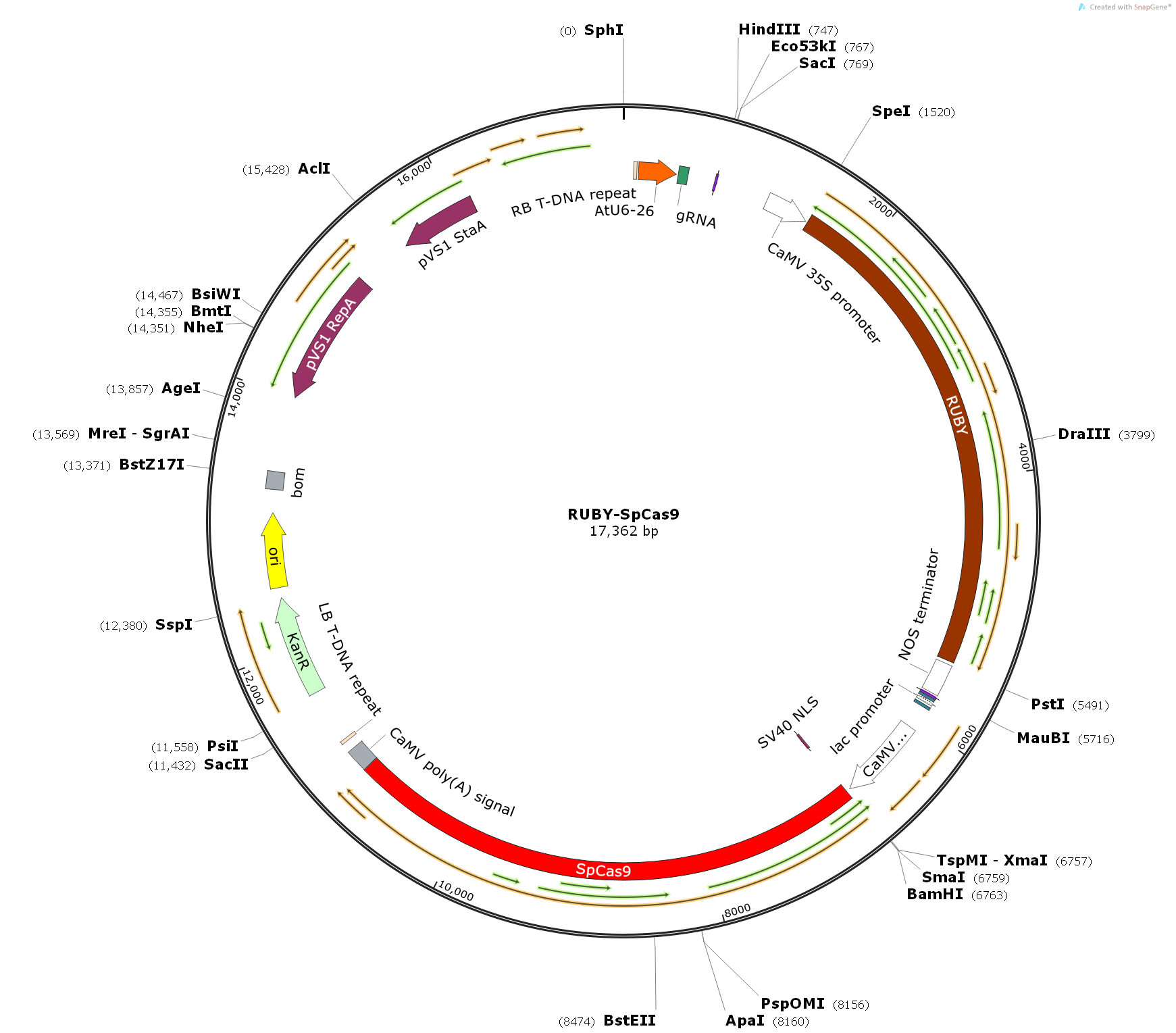

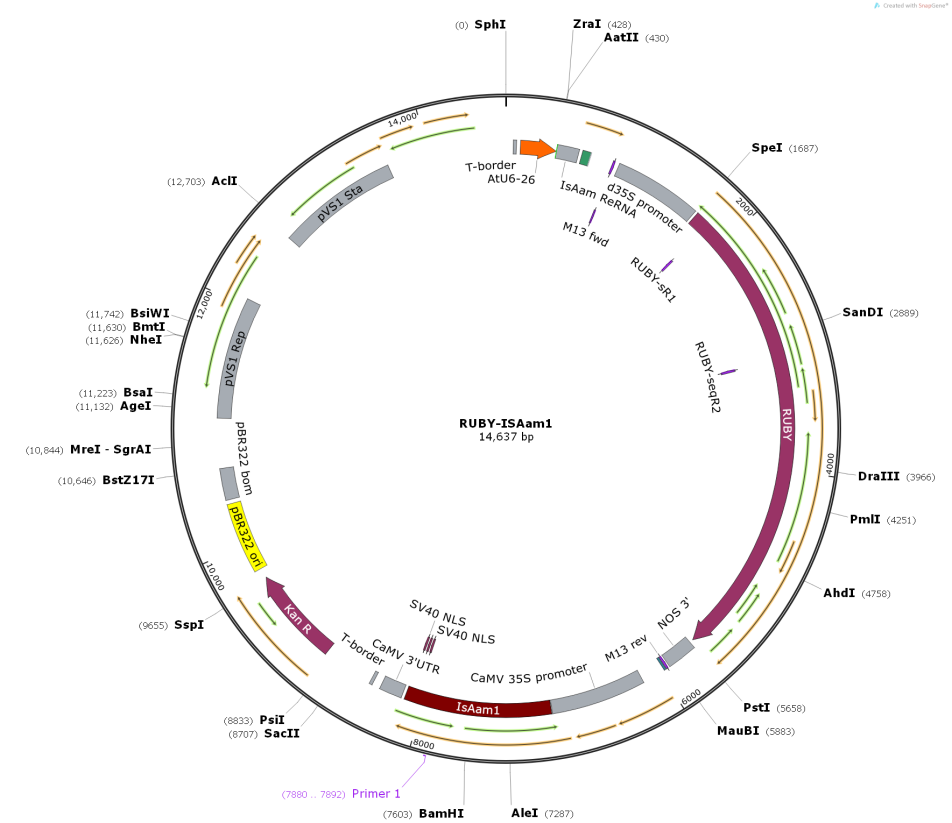
**

**Supplementary Figure 1. Maps of *RUBY-SpCas9* and *RUBY-ISAam1.***

**Supplementary table 1. Sequences of the *RUBY-SpCas9* and *RUBY-ISAam1.***

| **Name** | **Sequence (5’ to 3’)** | **Note** |
| --- | --- | --- |
| RUBY-SpCas9 | *cacatacaaatggacgaacggataaaccttttcacgcccttttaaatatccgttattctaataaacgctcttttctcttaggtttacccgccaatatatcctgtcaaacactgatagtttCTTTCCATTCGGAGTTTTTGTATCTTGTTTCATAGTTTGTCCCAGGATTAGAATGATTAGGCATCGAACCTTCAAGAATTTGATTGAATAAAACATCTTCATTCTTAAGATATGAAGATAATCTTCAAAAGGCCCCTGGGAATCTGAAAGAAGAGAAGCAGGCCCATTTATATGGGAAAGAACAATAGTATTTCTTATATAGGCCCATTTAAGTTGAAAACAATCTTCAAAAGTCCCACATCGCTTAGATAAGAAAACGAAGCTGAGTTTATATACAGCTAGAGTCGAAGTAGTGATTAGAGACCAAGGTCTCAGTTTTAGAGCTAGAAATAGCAAGTTAAAATAAGGCTAGTCCGTTATCAACTTGAAAAAGTGGCACCGAGTCGGTGCAAAAAATTTTTTTGCAAAATTTTCCAGAaaactgaaggcgggaaacgacaatctgatccaagctcaagctgctctagcattcgccattcaggctgcgcaactgttgggaagggcgatcggtgcgggcctcttcgctattacgccagctggcgaaagggggatgtgctgcaaggcgattaagttgggtaacgccagggttttcccagtcacgacgttgtaaaacgacggccagtgccaagcttgattaccaattcgagctccctacccctactccaaaaatgtcaaagatacagtctcagaagaccaaagggctattgagacttttcaacaaagggtaatttcgggaaacctcctcggattccattgcccagctatctgtcacttcatcgaaaggacagtagaaaaggaaggtggctcctacaaatgccatcattgcgataaaggaaaggctatcattcaagatgcctctgccgacagtggtcccaaagatggacccccacccacgaggagcatcgtggaaaaagaagacgttccaaccacgtcttcaaagcaagtggattgatgtgacatctccactgacgtaagggatgacgcacaatcccacccctactccaaaaatgtcaaagatacagtctcagaagaccaaagggctattgagacttttcaacaaagggtaatttcgggaaacctcctcggattccattgcccagctatctgtcacttcatcgaaaggacagtagaaaaggaaggtggctcctacaaatgccatcattgcgataaaggaaaggctatcattcaagatgcctctgccgacagtggtcccaaagatggacccccacccacgaggagcatcgtggaaaaagaagacgttccaaccacgtcttcaaagcaagtggattgatgtgacatctccactgacgtaagggatgacgcacaatcccactatccttcgcaagacccttcctctatataaggaagttcatttcatttggagaggacagcccagatcactagtatggatcatgcgaccctcgccatgatcctcgcgatctggttcatcagcttccacttcatcaagctgctgttctcccagcagaccaccaagctgcttccgccaggaccaaagccgcttccgatcatcggcaacatccttgaggtgggcaagaagccgcatcggtccttcgccaacctcgccaagattcacggcccactcatttccctcagactcggctctgtgaccaccatcgttgtgtcctctgccgacgtggccaaagagatgttcctcaagaaggatcacccgctctccaaccgcacgatcccgaatagtgttacagccggcgaccaccacaagctcaccatgtcttggctcccggtgtctccgaagtggcgcaacttccgcaagattaccgccgtgcatctgctctccccacagagactcgatgcctgccagacattcaggcacgccaaggtgcagcagctctacgagtacgttcaagagtgcgcccagaaaggccaggccgtggatattggcaaggccgcctttacgaccagcctcaacctcctcagcaagctgttcttcagcgtcgagctggcgcaccacaagtcccataccagccaagagttcaaagagctgatctggaacatcatggaagatataggcaagccgaactacgccgactacttcccgattctcggctgcgttgacccatctggcattagaagaaggctcgcctgctccttcgacaagctgatcgccgtgttccagggcatcatctgcgagagactcgccccagattcctccaccacaactaccaccaccaccgacgacgtgctcgatgtgctcctccagctgttcaagcagaacgagctgacgatgggcgagatcaaccacctcctcgtggacatcttcgacgccggcaccgataccacatcctccacattcgagtgggtgatgaccgagctgatccgcaatccagagatgatggaaaaggcccaagaggaaatcaagcaggtcctcggcaaggacaagcagatccaagagtccgacatcatcaacctgccgtacctccaggcgatcatcaaagagacactccgcctccatccgccgaccgtgttcttgctcccaagaaaggccgacaccgatgtcgagctgtacggctacatcgtgccgaaggatgcccagatcctcgtgaacctctgggccattggcagggacccaaacgcctggcagaacgccgatattttcagcccagagcgcttcatcggctgcgagatcgatgttaagggccgcgatttcggcctccttccatttggcgctggccgcagaatttgcccaggcatgaatctcgccatcaggatgctcaccctcatgctcgccacactcctccagttcttcaactggaagctcgaaggcgacatctccccgaaggacctcgacatggacgagaagttcggcattgcgctccaaaagaccaagccgctcaagctcatcccgattccgcgctaccaattgttgaattttgatttgttgaagttggctggagatgttgaatctaatcctggacctaagatgatgaacggcgaggacgccaacgaccagatgatcaaagagtccttcttcatcacccacggcaacccgatcctcaccgtcgaggatacacatccgctcaggccgttcttcgagacatggcgcgagaagattttctccaagaagccgaaggccatcctcatcatctccggccactgggagacagtgaagccaaccgtgaacgccgtgcacatcaacgacaccatccacgacttcgacgactacccagccgccatgtaccagttcaagtacccagctccaggcgagccagagcttgcgagaaaggtggaagagatcctcaagaagtccgggttcgagacagccgagacagaccaaaagaggggccttgatcacggcgcctgggttccactcatgctcatgtatccagaggcggacatcccggtgtgccagctctcagttcagccacatctcgacggcacctaccactacaatctcggcagagccctcgcgccgctcaagaatgatggcgtgctcattattggctccggcagcgccacacatccactcgatgagacaccgcactacttcgatggtgttgccccttgggccgctgccttcgattcttggcttaggaaggccctcatcaacggccgcttcgaggaagtgaacatctacgagagcaaggccccgaactggaagctcgcccatccatttccagagcacttctacccgctccacgttgtgctcggcgctgctggtgaaaagtggaaggccgagctgatccactcctcctgggatcatggcacactttgccacggctcctacaagttcacctccgcccaattgttgaattttgatttgttgaagttggctggagatgttgaatctaatcctggacctaccgccatcaagatgaacaccaacggcgagggcgagacacagcacatcctcatgatcccgttcatggcgcagggccacctcaggccatttctcgaactcgccatgttcctctacaagcgctcccacgtgatcatcaccctgctcacaactccgctcaacgccggcttcctcaggcacctccttcaccaccattcctactcctccagcggcatcaggatcgtcgagctgccattcaactccaccaaccacggactcccaccgggcatcgagaacaccgataagctcacactcccgctcgtggtgtccctcttccattccaccatcagcctcgatccgcacctccgcgattacatctccaggcatttcagcccagccaggccaccactctgcgtgatccatgatgtgttcctcggctgggttgaccaggtggccaaggatgtgggctctacaggcgtggtgttcacaacaggcggcgcttatggcacatccgcctacgtgtccatctggaacgatctcccgcaccagaactactccgacgaccaagagttcccgctgccaggcttcccagagaaccataagttccgcaggtcccagctccatcggttcctcagatatgccgacggctccgacgattggtccaagtatttccagccgcagctccgccagtccatgaagtcttttggctggctctgcaactccgtggaagagatcgagacactcggcttctccatcctccgcaactacaccaagctgccgatctggggcatcggcccacttattgcttccccagtgcagcactcctcctccgacaacaattcaacaggcgccgagttcgtgcagtggctcagcctcaaagagccggactccgtcctctacatctccttcggctcccagaacacgatcagcccgacgcagatgatggaactcgctgctggccttgagtcctccgagaagccattcctctgggtgatcagagccccgttcggcttcgacatcaacgaagagatgcgcccagagtggctgccagagggctttgaggaacgcatgaaggtgaagaaacagggcaagctcgtgtacaagctcggcccgcagcttgagatcctcaaccatgaatccatcggcggctttctcacccactgcggatggaacagcatccttgagtctcttcgcgagggcgttccgatgcttggatggccacttgctgccgagcaggcctacaacctcaagtacctcgaagatgagatgggcgtcgcggttgagcttgctagaggcctcgaaggcgagatctccaaagagaaggtcaagcgcatcgtcgagatgatccttgagcgcaacgagggctccaaaggctgggagatgaagaatcgcgccgtggaaatgggcaaaaagctcaaggacgccgtgaacgaggaaaaagagctgaagggctcctccgtgaaggcgatcgacgatttcctcgacgccgtcatgcaggccaaacttgagccaagcctccagtgatagtgaagatcttaaagcggccgcccggctgcagatcgttcaaacatttggcaataaagtttcttaagattgaatcctgttgccggtcttgcgatgattatcatataatttctgttgaattacgttaagcatgtaataattaacatgtaatgcatgacgttatttatgagatgggtttttatgattagagtcccgcaattatacatttaatacgcgatagaaaacaaaatatagcgcgcaaactaggataaattatcgcgcgcggtgtcatctatgttactagatcggattcgtaatcatggtcatagctgtttcctgtgtgaaattgttatccgctcacaattccacacaacatacgagccggaagcataaagtgtaaagcctggggtgcctaatgagtgagctaactcacattaattgcgttgcgctcactgcccgctttccagtcgggaaacctgtcgtgccagctgcattaatgaatcggccaacgcgcggggagaggcggtttgcgtattggctagagcagcttgccaacatggtggagcacgacactctcgtctactccaagaatatcaaagatacagtctcagaagaccaaagggctattgagacttttcaacaaagggtaatatcgggaaacctcctcggattccattgcccagctatctgtcacttcatcaaaaggacagtagaaaaggaaggtggcacctacaaatgccatcattgcgataaaggaaaggctatcgttcaagatgcctctgccgacagtggtcccaaagatggacccccacccacgaggagcatcgtggaaaaagaagacgttccaaccacgtcttcaaagcaagtggattgatgtgataacatggtggagcacgacactctcgtctactccaagaatatcaaagatacagtctcagaagaccaaagggctattgagacttttcaacaaagggtaatatcgggaaacctcctcggattccattgcccagctatctgtcacttcatcaaaaggacagtagaaaaggaaggtggcacctacaaatgccatcattgcgataaaggaaaggctatcgttcaagatgcctctgccgacagtggtcccaaagatggacccccacccacgaggagcatcgtggaaaaagaagacgttccaaccacgtcttcaaagcaagtggattgatgtgatatctccactgacgtaagggatgacgcacaatcccactatccttcgcaagaccttcctctatataaggaagttcatttcatttggagaggacacgctgaaatcaccagtctctCCCGGGGGATCCCCAATACTATGGCCCCAAAGAAGAAGCGCAAGGTCGACAAGAAGTACTCCATCGGCCTCGACATCGGCACCAATTCTGTTGGCTGGGCCGTGATCACCGACGAGTACAAGGTGCCGTCCAAGAAGTTCAAGGTCCTCGGCAACACCGACCGCCACTCCATCAAGAAGAATCTCATCGGCGCCCTGCTGTTCGACTCTGGCGAGACAGCCGAGGCTACAAGGCTCAAGAGGACCGCTAGACGCAGGTACACCAGGCGCAAGAACCGCATCTGCTACCTCCAAGAGATCTTCTCCAACGAGATGGCCAAGGTGGACGACAGCTTCTTCCACAGGCTCGAGGAGAGCTTCCTCGTCGAGGAGGACAAGAAGCACGAGCGCCATCCGATCTTCGGCAACATCGTGGATGAGGTGGCCTACCACGAGAAGTACCCGACCATCTACCACCTCCGCAAGAAGCTCGTCGACTCCACCGATAAGGCCGACCTCAGGCTCATCTACCTCGCCCTCGCCCACATGATCAAGTTCAGGGGCCACTTCCTCATCGAGGGCGACCTCAACCCGGACAACTCCGATGTGGACAAGCTGTTCATCCAGCTCGTGCAGACCTACAACCAGCTGTTCGAGGAGAACCCGATCAACGCCTCTGGCGTTGACGCCAAGGCTATTCTCTCTGCCAGGCTCTCTAAGTCCCGCAGGCTCGAGAATCTGATCGCCCAACTTCCGGGCGAGAAGAAGAATGGCCTCTTCGGCAACCTGATCGCCCTCTCTCTTGGCCTCACCCCGAACTTCAAGTCCAACTTCGACCTCGCCGAGGACGCCAAGCTCCAGCTTTCCAAGGACACCTACGACGACGACCTCGACAATCTCCTCGCCCAGATTGGCGATCAGTACGCCGATCTGTTCCTCGCCGCCAAGAATCTCTCCGACGCCATCCTCCTCAGCGACATCCTCAGGGTGAACACCGAGATCACCAAGGCCCCACTCTCCGCCTCCATGATCAAGAGGTACGACGAGCACCACCAGGACCTCACACTCCTCAAGGCCCTCGTGAGACAGCAGCTCCCAGAGAAGTACAAGGAGATCTTCTTCGACCAGTCCAAGAACGGCTACGCCGGCTACATCGATGGCGGCGCTTCTCAAGAGGAGTTCTACAAGTTCATCAAGCCGATCCTCGAGAAGATGGACGGCACCGAGGAGCTGCTCGTGAAGCTCAATAGAGAGGACCTCCTCCGCAAGCAGCGCACCTTCGATAATGGCTCCATCCCGCACCAGATCCACCTCGGCGAGCTTCATGCTATCCTCCGCAGGCAAGAGGACTTCTACCCGTTCCTCAAGGACAACCGCGAGAAGATTGAGAAGATCCTCACCTTCCGCATCCCGTACTACGTGGGCCCGCTCGCCAGGGGCAACTCCAGGTTCGCCTGGATGACCAGAAAGTCCGAGGAGACAATCACCCCCTGGAACTTCGAGGAGGTGGTGGATAAGGGCGCCTCTGCCCAGTCTTTCATCGAGCGCATGACCAACTTCGACAAGAACCTCCCGAACGAGAAGGTGCTCCCGAAGCACTCACTCCTCTACGAGTACTTCACCGTGTACAACGAGCTGACCAAGGTGAAGTACGTGACCGAGGGGATGAGGAAGCCAGCTTTCCTTAGCGGCGAGCAAAAGAAGGCCATCGTCGACCTGCTGTTCAAGACCAACCGCAAGGTGACCGTGAAGCAGCTCAAGGAGGACTACTTCAAGAAAATCGAGTGCTTCGACTCCGTCGAGATCTCCGGCGTCGAGGATAGGTTCAATGCCTCCCTCGGGACCTACCACGACCTCCTCAAGATTATCAAGGACAAGGACTTCCTCGACAACGAGGAGAACGAGGACATCCTCGAGGACATCGTGCTCACCCTCACCCTCTTCGAGGACCGCGAGATGATCGAGGAGCGCCTCAAGACATACGCCCACCTCTTCGACGACAAGGTGATGAAGCAGCTGAAGCGCAGGCGCTATACCGGCTGGGGCAGGCTCTCTAGGAAGCTCATCAACGGCATCCGCGACAAGCAGTCCGGCAAGACGATCCTCGACTTCCTCAAGTCCGACGGCTTCGCCAACCGCAACTTCATGCAGCTCATCCACGACGACTCCCTCACCTTCAAGGAGGACATCCAAAAGGCCCAGGTGTCCGGCCAAGGCGATTCCCTCCATGAGCATATCGCCAATCTCGCCGGCTCCCCGGCTATCAAGAAGGGCATTCTCCAGACCGTGAAGGTGGTGGACGAGCTGGTGAAGGTGATGGGCAGGCACAAGCCAGAGAACATCGTGATCGAGATGGCCCGCGAGAACCAGACCACACAGAAGGGCCAAAAGAACTCCCGCGAGCGCATGAAGAGGATCGAGGAGGGCATTAAGGAGCTGGGCTCCCAGATCCTCAAGGAGCACCCAGTCGAGAACACCCAGCTCCAGAACGAGAAGCTCTACCTCTACTACCTCCAGAACGGCCGCGACATGTACGTGGACCAAGAGCTGGACATCAACCGCCTCTCCGACTACGACGTGGACCATATTGTGCCGCAGTCCTTCCTGAAGGACGACTCCATCGACAACAAGGTGCTCACCCGCTCCGACAAGAACAGGGGCAAGTCCGATAACGTGCCGTCCGAAGAGGTCGTCAAGAAGATGAAGAACTACTGGCGCCAGCTCCTCAACGCCAAGCTCATCACCCAGAGGAAGTTCGACAACCTCACCAAGGCCGAGAGAGGCGGCCTTTCCGAGCTTGATAAGGCCGGCTTCATCAAGCGCCAGCTCGTCGAGACACGCCAGATCACAAAGCACGTGGCCCAGATCCTCGACTCCCGCATGAACACCAAGTACGACGAGAACGACAAGCTCATCCGCGAGGTGAAGGTCATCACCCTCAAGTCCAAGCTCGTGTCCGACTTCCGCAAGGACTTCCAGTTCTACAAGGTGCGCGAGATCAACAACTACCACCACGCCCACGACGCCTACCTCAATGCCGTGGTGGGCACAGCCCTCATCAAGAAGTACCCAAAGCTCGAGTCCGAGTTCGTGTACGGCGACTACAAGGTGTACGACGTGCGCAAGATGATCGCCAAGTCCGAGCAAGAGATCGGCAAGGCGACCGCCAAGTACTTCTTCTACTCCAACATCATGAATTTCTTCAAGACCGAGATCACGCTCGCCAACGGCGAGATTAGGAAGAGGCCGCTCATCGAGACAAACGGCGAGACAGGCGAGATCGTGTGGGACAAGGGCAGGGATTTCGCCACAGTGCGCAAGGTGCTCTCCATGCCGCAAGTGAACATCGTGAAGAAGACCGAGGTTCAGACCGGCGGCTTCTCCAAGGAGTCCATCCTCCCAAAGCGCAACTCCGACAAGCTGATCGCCCGCAAGAAGGACTGGGACCCGAAGAAGTATGGCGGCTTCGATTCTCCGACCGTGGCCTACTCTGTGCTCGTGGTTGCCAAGGTCGAGAAGGGCAAGAGCAAGAAGCTCAAGTCCGTCAAGGAGCTGCTGGGCATCACGATCATGGAGCGCAGCAGCTTCGAGAAGAACCCAATCGACTTCCTCGAGGCCAAGGGCTACAAGGAGGTGAAGAAGGACCTCATCATCAAGCTCCCGAAGTACAGCCTCTTCGAGCTTGAGAACGGCCGCAAGAGAATGCTCGCCTCTGCTGGCGAGCTTCAGAAGGGCAACGAGCTTGCTCTCCCGTCCAAGTACGTGAACTTCCTCTACCTCGCCTCCCACTACGAGAAGCTCAAGGGCTCCCCAGAGGACAACGAGCAAAAGCAGCTGTTCGTCGAGCAGCACAAGCACTACCTCGACGAGATCATCGAGCAGATCTCCGAGTTCTCCAAGCGCGTGATCCTCGCCGATGCCAACCTCGATAAGGTGCTCAGCGCCTACAACAAGCACCGCGATAAGCCAATTCGCGAGCAGGCCGAGAACATCATCCACCTCTTCACCCTCACCAACCTCGGCGCTCCAGCCGCCTTCAAGTACTTCGACACCACCATCGACCGCAAGCGCTACACCTCTACCAAGGAGGTTCTCGACGCCACCCTCATCCACCAGTCTATCACAGGCCTCTACGAGACACGCATCGACCTCTCACAACTCGGCGGCGATTGActcgagtttctccataataatgtgtgagtagttcccagataagggaattagggttcctatagggtttcgctcatgtgttgagcatataagaaacccttagtatgtatttgtatttgtaaaatacttctatcaataaaatttctaattcctaaaaccaaaatccagtactaaaatccagatcccccgaattaattcggcgttaattcagtacattaaaaacgtccgcaatgtgttattaagttgtctaagcgtcaatttgtttacaccacaatatatcctgccaccagccagccaacagctccccgaccggcagctcggcacaaaatcaccactcgatacaggcagcccatcagtccgggacggcgtcagcgggagagccgttgtaaggcggcagactttgctcatgttaccgatgctattcggaagaacggcaactaagctgccgggtttgaaacacggatgatctcgcggagggtagcatgttgattgtaacgatgacagagcgttgctgcctgtgatcaccgcggtttcaaaatcggctccgtcgatactatgttatacgccaactttgaaaacaactttgaaaaagctgttttctggtatttaaggttttagaatgcaaggaacagtgaattggagttcgtcttgttataattagcttcttggggtatctttaaatactgtagaaaagaggaaggaaataataaatggctaaaatgagaatatcaccggaattgaaaaaactgatcgaaaaataccgctgcgtaaaagatacggaaggaatgtctcctgctaaggtatataagctggtgggagaaaatgaaaacctatatttaaaaatgacggacagccggtataaagggaccacctatgatgtggaacgggaaaaggacatgatgctatggctggaaggaaagctgcctgttccaaaggtcctgcactttgaacggcatgatggctggagcaatctgctcatgagtgaggccgatggcgtcctttgctcggaagagtatgaagatgaacaaagccctgaaaagattatcgagctgtatgcggagtgcatcaggctctttcactccatcgacatatcggattgtccctatacgaatagcttagacagccgcttagccgaattggattacttactgaataacgatctggccgatgtggattgcgaaaactgggaagaagacactccatttaaagatccgcgcgagctgtatgattttttaaagacggaaaagcccgaagaggaacttgtcttttcccacggcgacctgggagacagcaacatctttgtgaaagatggcaaagtaagtggctttattgatcttgggagaagcggcagggcggacaagtggtatgacattgccttctgcgtccggtcgatcagggaggatatcggggaagaacagtatgtcgagctattttttgacttactggggatcaagcctgattgggagaaaataaaatattatattttactggatgaattgttttagtacctagaatgcatgaccaaaatcccttaacgtgagttttcgttccactgagcgtcagaccccgtagaaaagatcaaaggatcttcttgagatcctttttttctgcgcgtaatctgctgcttgcaaacaaaaaaaccaccgctaccagcggtggtttgtttgccggatcaagagctaccaactctttttccgaaggtaactggcttcagcagagcgcagataccaaatactgtccttctagtgtagccgtagttaggccaccacttcaagaactctgtagcaccgcctacatacctcgctctgctaatcctgttaccagtggctgctgccagtggcgataagtcgtgtcttaccgggttggactcaagacgatagttaccggataaggcgcagcggtcgggctgaacggggggttcgtgcacacagcccagcttggagcgaacgacctacaccgaactgagatacctacagcgtgagctatgagaaagcgccacgcttcccgaagggagaaaggcggacaggtatccggtaagcggcagggtcggaacaggagagcgcacgagggagcttccagggggaaacgcctggtatctttatagtcctgtcgggtttcgccacctctgacttgagcgtcgatttttgtgatgctcgtcaggggggcggagcctatggaaaaacgccagcaacgcggcctttttacggttcctggccttttgctggccttttgctcacatgttctttcctgcgttatcccctgattctgtggataaccgtattaccgcctttgagtgagctgataccgctcgccgcagccgaacgaccgagcgcagcgagtcagtgagcgaggaagcggaagagcgcctgatgcggtattttctccttacgcatctgtgcggtatttcacaccgcatatggtgcactctcagtacaatctgctctgatgccgcatagttaagccagtatacactccgctatcgctacgtgactgggtcatggctgcgccccgacacccgccaacacccgctgacgcgccctgacgggcttgtctgctcccggcatccgcttacagacaagctgtgaccgtctccgggagctgcatgtgtcagaggttttcaccgtcatcaccgaaacgcgcgaggcagggtgccttgatgtgggcgccggcggtcgagtggcgacggcgcggcttgtccgcgccctggtagattgcctggccgtaggccagccatttttgagcggccagcggccgcgataggccgacgcgaagcggcggggcgtagggagcgcagcgaccgaagggtaggcgctttttgcagctcttcggctgtgcgctggccagacagttatgcacaggccaggcgggttttaagagttttaataagttttaaagagttttaggcggaaaaatcgccttttttctcttttatatcagtcacttacatgtgtgaccggttcccaatgtacggctttgggttcccaatgtacgggttccggttcccaatgtacggctttgggttcccaatgtacgtgctatccacaggaaagagaccttttcgacctttttcccctgctagggcaatttgccctagcatctgctccgtacattaggaaccggcggatgcttcgccctcgatcaggttgcggtagcgcatgactaggatcgggccagcctgccccgcctcctccttcaaatcgtactccggcaggtcatttgacccgatcagcttgcgcacggtgaaacagaacttcttgaactctccggcgctgccactgcgttcgtagatcgtcttgaacaaccatctggcttctgccttgcctgcggcgcggcgtgccaggcggtagagaaaacggccgatgccgggatcgatcaaaaagtaatcggggtgaaccgtcagcacgtccgggttcttgccttctgtgatctcgcggtacatccaatcagctagctcgatctcgatgtactccggccgcccggtttcgctctttacgatcttgtagcggctaatcaaggcttcaccctcggataccgtcaccaggcggccgttcttggccttcttcgtacgctgcatggcaacgtgcgtggtgtttaaccgaatgcaggtttctaccaggtcgtctttctgctttccgccatcggctcgccggcagaacttgagtacgtccgcaacgtgtggacggaacacgcggccgggcttgtctcccttcccttcccggtatcggttcatggattcggttagatgggaaaccgccatcagtaccaggtcgtaatcccacacactggccatgccggccggccctgcggaaacctctacgtgcccgtctggaagctcgtagcggatcacctcgccagctcgtcggtcacgcttcgacagacggaaaacggccacgtccatgatgctgcgactatcgcgggtgcccacgtcatagagcatcggaacgaaaaaatctggttgctcgtcgcccttgggcggcttcctaatcgacggcgcaccggctgccggcggttgccgggattctttgcggattcgatcagcggccgcttgccacgattcaccggggcgtgcttctgcctcgatgcgttgccgctgggcggcctgcgcggccttcaacttctccaccaggtcatcacccagcgccgcgccgatttgtaccgggccggatggtttgcgaccgtcacgccgattcctcgggcttgggggttccagtgccattgcagggccggcagacaacccagccgcttacgcctggccaaccgcccgttcctccacacatggggcattccacggcgtcggtgcctggttgttcttgattttccatgccgcctcctttagccgctaaaattcatctactcatttattcatttgctcatttactctggtagctgcgcgatgtattcagatagcagctcggtaatggtcttgccttggcgtaccgcgtacatcttcagcttggtgtgatcctccgccggcaactgaaagttgacccgcttcatggctggcgtgtctgccaggctggccaacgttgcagccttgctgctgcgtgcgctcggacggccggcacttagcgtgtttgtgcttttgctcattttctctttacctcattaactcaaatgagttttgatttaatttcagcggccagcgcctggacctcgcgggcagcgtcgccctcgggttctgattcaagaacggttgtgccggcggcggcagtgcctgggtagctcacgcgctgcgtgatacgggactcaagaatgggcagctcgtacccggccagcgcctcggcaacctcaccgccgatgcgcgtgcctttgatcgcccgcgacacgacaaaggccgcttgtagccttccatccgtgacctcaatgcgctgcttaaccagctccaccaggtcggcggtggcccatatgtcgtaagggcttggctgcaccggaatcagcacgaagtcggctgccttgatcgcggacacagccaagtccgccgcctggggcgctccgtcgatcactacgaagtcgcgccggccgatggccttcacgtcgcggtcaatcgtcgggcggtcgatgccgacaacggttagcggttgatcttcccgcacggccgcccaatcgcgggcactgccctggggatcggaatcgactaacagaacatcggccccggcgagttgcagggcgcgggctagatgggttgcgatggtcgtcttgcctgacccgcctttctggttaagtacagcgataaccttcatgcgttccccttgcgtatttgtttatttactcatcgcatcatatacgcagcgaccgcatgacgcaagctgttttactcaaatacacatcacctttttagacggcggcgctcggtttcttcagcggccaagctggccggccaggccgccagcttggcatcagacaaaccggccaggatttcatgcagccgcacggttgagacgtgcgcgggcggctcgaacacgtacccggccgcgatcatctccgcctcgatctcttcggtaatgaaaaacggttcgtcctggccgtcctggtgcggtttcatgcttgttcctcttggcgttcattctcggcggccgccagggcgtcggcctcggtcaatgcgtcctcacggaaggcaccgcgccgcctggcctcggtgggcgtcacttcctcgctgcgctcaagtgcgcggtacagggtcgagcgatgcacgccaagcagtgcagccgcctctttcacggtgcggccttcctggtcgatcagctcgcgggcgtgcgcgatctgtgccggggtgagggtagggcgggggccaaacttcacgcctcgggccttggcggcctcgcgcccgctccgggtgcggtcgatgattagggaacgctcgaactcggcaatgccggcgaacacggtcaacaccatgcggccggccggcgtggtggtgtcggcccacggctctgccaggctacgcaggcccgcgccggcctcctggatgcgctcggcaatgtccagtaggtcgcgggtgctgcgggccaggcggtctagcctggtcactgtcacaacgtcgccagggcgtaggtggtcaagcatcctggccagctccgggcggtcgcgcctggtgccggtgatcttctcggaaaacagcttggtgcagccggccgcgtgcagttcggcccgttggttggtcaagtcctggtcgtcggtgctgacgcgggcatagcccagcaggccagcggcggcgctcttgttcatggcgtaatgtctccggttctagtcgcaagtattctactttatgcgactaaaacacgcgacaagaaaacgccaggaaaagggcagggcggcagcctgtcgcgtaacttaggacttgtgcgacatgtcgttttcagaagacggctgcactgaacgtcagaagccgactgcactatagcagcggaggggttggatcaaagtactttgatcccgaggggaaccctgtggttggcatg* | *AtU6-*gRNA scaffold-Ploy T-*CaMV 35S promoter-SV40 NLS SpCas9-CaMV 3'UTR* |
| *RUBY-ISAam1* | *cacatacaaatggacgaacggataaaccttttcacgcccttttaaatatccgttattctaataaacgctcttttctcttaggtttacccgccaatatatcctgtcaaacactgatagtttCTTTCCATTCGGAGTTTTTGTATCTTGTTTCATAGTTTGTCCCAGGATTAGAATGATTAGGCATCGAACCTTCAAGAATTTGATTGAATAAAACATCTTCATTCTTAAGATATGAAGATAATCTTCAAAAGGCCCCTGGGAATCTGAAAGAAGAGAAGCAGGCCCATTTATATGGGAAAGAACAATAGTATTTCTTATATAGGCCCATTTAAGTTGAAAACAATCTTCAAAAGTCCCACATCGCTTAGATAAGAAAACGAAGCTGAGTTTATATACAGCTAGAGTCGAAGTAGTGATTgGACAGGGACGTCAATGCGGCAATCAATATCAAACATGAGGGCATGAAACGATTAGCAATAGCCTAACTTGTCCTCGAACCGTGGGACACACGGGGATCGCTCAGTCAACTTCCCGTCATGAGATGGGATTACCTGAGAAGCCCCCACCTCTAAGCGAAGCGTAGGTGGTGGGAGCATGTCACGCTCGCAGGTGAACACAACACCTGCACACTTTTTTTAGCATGACGGCGCACCCTggccggcatggtcccagcctcctcgctggcgccggctgggcaacatgcttcggcatggcgaatgggacTTTTTTTaaactgaaggcgggaaacgacaatctgatccaagctcaagctgctctagcattcgccattcaggctgcgcaactgttgggaagggcgatcggtgcgggcctcttcgctattacgccagctggcgaaagggggatgtgctgcaaggcgattaagttgggtaacgccagggttttcccagtcacgacgttgtaaaacgacggccagtgccaagcttgattaccaattcgagctccctacccctactccaaaaatgtcaaagatacagtctcagaagaccaaagggctattgagacttttcaacaaagggtaatttcgggaaacctcctcggattccattgcccagctatctgtcacttcatcgaaaggacagtagaaaaggaaggtggctcctacaaatgccatcattgcgataaaggaaaggctatcattcaagatgcctctgccgacagtggtcccaaagatggacccccacccacgaggagcatcgtggaaaaagaagacgttccaaccacgtcttcaaagcaagtggattgatgtgacatctccactgacgtaagggatgacgcacaatcccacccctactccaaaaatgtcaaagatacagtctcagaagaccaaagggctattgagacttttcaacaaagggtaatttcgggaaacctcctcggattccattgcccagctatctgtcacttcatcgaaaggacagtagaaaaggaaggtggctcctacaaatgccatcattgcgataaaggaaaggctatcattcaagatgcctctgccgacagtggtcccaaagatggacccccacccacgaggagcatcgtggaaaaagaagacgttccaaccacgtcttcaaagcaagtggattgatgtgacatctccactgacgtaagggatgacgcacaatcccactatccttcgcaagacccttcctctatataaggaagttcatttcatttggagaggacagcccagatcactagtatggatcatgcgaccctcgccatgatcctcgcgatctggttcatcagcttccacttcatcaagctgctgttctcccagcagaccaccaagctgcttccgccaggaccaaagccgcttccgatcatcggcaacatccttgaggtgggcaagaagccgcatcggtccttcgccaacctcgccaagattcacggcccactcatttccctcagactcggctctgtgaccaccatcgttgtgtcctctgccgacgtggccaaagagatgttcctcaagaaggatcacccgctctccaaccgcacgatcccgaatagtgttacagccggcgaccaccacaagctcaccatgtcttggctcccggtgtctccgaagtggcgcaacttccgcaagattaccgccgtgcatctgctctccccacagagactcgatgcctgccagacattcaggcacgccaaggtgcagcagctctacgagtacgttcaagagtgcgcccagaaaggccaggccgtggatattggcaaggccgcctttacgaccagcctcaacctcctcagcaagctgttcttcagcgtcgagctggcgcaccacaagtcccataccagccaagagttcaaagagctgatctggaacatcatggaagatataggcaagccgaactacgccgactacttcccgattctcggctgcgttgacccatctggcattagaagaaggctcgcctgctccttcgacaagctgatcgccgtgttccagggcatcatctgcgagagactcgccccagattcctccaccacaactaccaccaccaccgacgacgtgctcgatgtgctcctccagctgttcaagcagaacgagctgacgatgggcgagatcaaccacctcctcgtggacatcttcgacgccggcaccgataccacatcctccacattcgagtgggtgatgaccgagctgatccgcaatccagagatgatggaaaaggcccaagaggaaatcaagcaggtcctcggcaaggacaagcagatccaagagtccgacatcatcaacctgccgtacctccaggcgatcatcaaagagacactccgcctccatccgccgaccgtgttcttgctcccaagaaaggccgacaccgatgtcgagctgtacggctacatcgtgccgaaggatgcccagatcctcgtgaacctctgggccattggcagggacccaaacgcctggcagaacgccgatattttcagcccagagcgcttcatcggctgcgagatcgatgttaagggccgcgatttcggcctccttccatttggcgctggccgcagaatttgcccaggcatgaatctcgccatcaggatgctcaccctcatgctcgccacactcctccagttcttcaactggaagctcgaaggcgacatctccccgaaggacctcgacatggacgagaagttcggcattgcgctccaaaagaccaagccgctcaagctcatcccgattccgcgctaccaattgttgaattttgatttgttgaagttggctggagatgttgaatctaatcctggacctaagatgatgaacggcgaggacgccaacgaccagatgatcaaagagtccttcttcatcacccacggcaacccgatcctcaccgtcgaggatacacatccgctcaggccgttcttcgagacatggcgcgagaagattttctccaagaagccgaaggccatcctcatcatctccggccactgggagacagtgaagccaaccgtgaacgccgtgcacatcaacgacaccatccacgacttcgacgactacccagccgccatgtaccagttcaagtacccagctccaggcgagccagagcttgcgagaaaggtggaagagatcctcaagaagtccgggttcgagacagccgagacagaccaaaagaggggccttgatcacggcgcctgggttccactcatgctcatgtatccagaggcggacatcccggtgtgccagctctcagttcagccacatctcgacggcacctaccactacaatctcggcagagccctcgcgccgctcaagaatgatggcgtgctcattattggctccggcagcgccacacatccactcgatgagacaccgcactacttcgatggtgttgccccttgggccgctgccttcgattcttggcttaggaaggccctcatcaacggccgcttcgaggaagtgaacatctacgagagcaaggccccgaactggaagctcgcccatccatttccagagcacttctacccgctccacgttgtgctcggcgctgctggtgaaaagtggaaggccgagctgatccactcctcctgggatcatggcacactttgccacggctcctacaagttcacctccgcccaattgttgaattttgatttgttgaagttggctggagatgttgaatctaatcctggacctaccgccatcaagatgaacaccaacggcgagggcgagacacagcacatcctcatgatcccgttcatggcgcagggccacctcaggccatttctcgaactcgccatgttcctctacaagcgctcccacgtgatcatcaccctgctcacaactccgctcaacgccggcttcctcaggcacctccttcaccaccattcctactcctccagcggcatcaggatcgtcgagctgccattcaactccaccaaccacggactcccaccgggcatcgagaacaccgataagctcacactcccgctcgtggtgtccctcttccattccaccatcagcctcgatccgcacctccgcgattacatctccaggcatttcagcccagccaggccaccactctgcgtgatccatgatgtgttcctcggctgggttgaccaggtggccaaggatgtgggctctacaggcgtggtgttcacaacaggcggcgcttatggcacatccgcctacgtgtccatctggaacgatctcccgcaccagaactactccgacgaccaagagttcccgctgccaggcttcccagagaaccataagttccgcaggtcccagctccatcggttcctcagatatgccgacggctccgacgattggtccaagtatttccagccgcagctccgccagtccatgaagtcttttggctggctctgcaactccgtggaagagatcgagacactcggcttctccatcctccgcaactacaccaagctgccgatctggggcatcggcccacttattgcttccccagtgcagcactcctcctccgacaacaattcaacaggcgccgagttcgtgcagtggctcagcctcaaagagccggactccgtcctctacatctccttcggctcccagaacacgatcagcccgacgcagatgatggaactcgctgctggccttgagtcctccgagaagccattcctctgggtgatcagagccccgttcggcttcgacatcaacgaagagatgcgcccagagtggctgccagagggctttgaggaacgcatgaaggtgaagaaacagggcaagctcgtgtacaagctcggcccgcagcttgagatcctcaaccatgaatccatcggcggctttctcacccactgcggatggaacagcatccttgagtctcttcgcgagggcgttccgatgcttggatggccacttgctgccgagcaggcctacaacctcaagtacctcgaagatgagatgggcgtcgcggttgagcttgctagaggcctcgaaggcgagatctccaaagagaaggtcaagcgcatcgtcgagatgatccttgagcgcaacgagggctccaaaggctgggagatgaagaatcgcgccgtggaaatgggcaaaaagctcaaggacgccgtgaacgaggaaaaagagctgaagggctcctccgtgaaggcgatcgacgatttcctcgacgccgtcatgcaggccaaacttgagccaagcctccagtgatagtgaagatcttaaagcggccgcccggctgcagatcgttcaaacatttggcaataaagtttcttaagattgaatcctgttgccggtcttgcgatgattatcatataatttctgttgaattacgttaagcatgtaataattaacatgtaatgcatgacgttatttatgagatgggtttttatgattagagtcccgcaattatacatttaatacgcgatagaaaacaaaatatagcgcgcaaactaggataaattatcgcgcgcggtgtcatctatgttactagatcggattcgtaatcatggtcatagctgtttcctgtgtgaaattgttatccgctcacaattccacacaacatacgagccggaagcataaagtgtaaagcctggggtgcctaatgagtgagctaactcacattaattgcgttgcgctcactgcccgctttccagtcgggaaacctgtcgtgccagctgcattaatgaatcggccaacgcgcggggagaggcggtttgcgtattggctagagcagcttgccaacatggtggagcacgacactctcgtctactccaagaatatcaaagatacagtctcagaagaccaaagggctattgagacttttcaacaaagggtaatatcgggaaacctcctcggattccattgcccagctatctgtcacttcatcaaaaggacagtagaaaaggaaggtggcacctacaaatgccatcattgcgataaaggaaaggctatcgttcaagatgcctctgccgacagtggtcccaaagatggacccccacccacgaggagcatcgtggaaaaagaagacgttccaaccacgtcttcaaagcaagtggattgatgtgataacatggtggagcacgacactctcgtctactccaagaatatcaaagatacagtctcagaagaccaaagggctattgagacttttcaacaaagggtaatatcgggaaacctcctcggattccattgcccagctatctgtcacttcatcaaaaggacagtagaaaaggaaggtggcacctacaaatgccatcattgcgataaaggaaaggctatcgttcaagatgcctctgccgacagtggtcccaaagatggacccccacccacgaggagcatcgtggaaaaagaagacgttccaaccacgtcttcaaagcaagtggattgatgtgatatctccactgacgtaagggatgacgcacaatcccactatccttcgcaagaccttcctctatataaggaagttcatttcatttggagaggacacgctgaaatcaccagtctctctctacaaatctatctctctcgagATGGTGAATAAGTCTTACAAGTTTAGACTCTACCCAACTAAGGAGCAGGAGCAACTCCTGGCCAAGACCTTCGGCTGCGTGAGGTTCGTGTACAACAAGATGCTCGAGGAGCGCATCCAGATCTACGAGAAGTTCAAGGACGACAAGGAGGCCCTCAAGAAGCAGACCTTCCCGACCCCAGCCAAGTACAAGAAGGAGTTCCCGTGGCTCAAGGAGGTCGATTCCCTGGCCCTCGCCAACGCGCAGTTGAACCTCCAGAAGGCGTTCCAGAACTTCTTCAGCGGCAGGGCGGGCTTCCCAAAGTTCAAGAACCGCAAGGCCAAGCAGAGCTACACCACCAACGTGGTGAACGGCAACATCCAGCTCTCCGACGGCTACATCAAGCTCCCGAAGCTCAAGTGGGTGAAGTTCAAGCAGCACAGGGAGATCCCGGCCCACCACATCATCAAGGCCTGCACCATCAAAAAGACCAAGACCGGCAAGTACTACGTGAGCATCCTGACAGAGTACGAGCACCAGCCGGTGCCAAAGGAGATCCAGACCGTGGTGGGCCTCGACTTCAGCATGAACGGCCTCTTCGTGGATAGCGAGGGCAAGAGGGCCAACTACCCGCGCTTCTACAGGCAGGCCCTCGAGAAGCTCGCCAAGGAGCAGAGGATCCTCTCCCGCCGCAAGAAGGGCAGCAATCGCTGGCATAAGCAGAGGCTCAAGGTGGCCAAGCTCCACGAGAAGATCGCGAATCAGCGCAAGGACTTCCTGCACAAGAAGTCCTACGAGCTCGCCAAGCAGTACGACTGCGTGGTCATCGAGAACCTCAACATGAAGGGCATGAGCCAGGTGCTGAACTTCGGCAAGTCCGTGCACGATAACGGCTGGGGCATGTTCACCACCTTCCTCCAGTACAAGCTCGAGGAGCAGGGCAAGAAGCTGATCAAGATCGACAAGTGGTTCCCGAGCAGCAAGACCTGCTCCTGCTGCGACCAGGTGAAGGAGAGCCTCTCCCTCAGCGAGAGGACCTTCCGGTGCGAGTGCGGCTTCGAGTCCGATAGGGATGTGAACGCCGCCATCAACATCAAGCACGAGGGCATGAAGCGCCTCGCCATCGCGTCCGGCGGCAGCCCTAAGAAGAAGAGGAAGGTGTCCGGCGGCTCCCCGAAGAAGAAGCGCAAGGTGTCCGGCGGCTCCCCGAAGAAGAAGCGCAAGGTGTGAAAGCTTGGCGTAATCATGGTCATAGCctcgagtttctccataataatgtgtgagtagttcccagataagggaattagggttcctatagggtttcgctcatgtgttgagcatataagaaacccttagtatgtatttgtatttgtaaaatacttctatcaataaaatttctaattcctaaaaccaaaatccagtactaaaatccagatcccccgaattaattcggcgttaattcagtacattaaaaacgtccgcaatgtgttattaagttgtctaagcgtcaatttgtttacaccacaatatatcctgccaccagccagccaacagctccccgaccggcagctcggcacaaaatcaccactcgatacaggcagcccatcagtccgggacggcgtcagcgggagagccgttgtaaggcggcagactttgctcatgttaccgatgctattcggaagaacggcaactaagctgccgggtttgaaacacggatgatctcgcggagggtagcatgttgattgtaacgatgacagagcgttgctgcctgtgatcaccgcggtttcaaaatcggctccgtcgatactatgttatacgccaactttgaaaacaactttgaaaaagctgttttctggtatttaaggttttagaatgcaaggaacagtgaattggagttcgtcttgttataattagcttcttggggtatctttaaatactgtagaaaagaggaaggaaataataaatggctaaaatgagaatatcaccggaattgaaaaaactgatcgaaaaataccgctgcgtaaaagatacggaaggaatgtctcctgctaaggtatataagctggtgggagaaaatgaaaacctatatttaaaaatgacggacagccggtataaagggaccacctatgatgtggaacgggaaaaggacatgatgctatggctggaaggaaagctgcctgttccaaaggtcctgcactttgaacggcatgatggctggagcaatctgctcatgagtgaggccgatggcgtcctttgctcggaagagtatgaagatgaacaaagccctgaaaagattatcgagctgtatgcggagtgcatcaggctctttcactccatcgacatatcggattgtccctatacgaatagcttagacagccgcttagccgaattggattacttactgaataacgatctggccgatgtggattgcgaaaactgggaagaagacactccatttaaagatccgcgcgagctgtatgattttttaaagacggaaaagcccgaagaggaacttgtcttttcccacggcgacctgggagacagcaacatctttgtgaaagatggcaaagtaagtggctttattgatcttgggagaagcggcagggcggacaagtggtatgacattgccttctgcgtccggtcgatcagggaggatatcggggaagaacagtatgtcgagctattttttgacttactggggatcaagcctgattgggagaaaataaaatattatattttactggatgaattgttttagtacctagaatgcatgaccaaaatcccttaacgtgagttttcgttccactgagcgtcagaccccgtagaaaagatcaaaggatcttcttgagatcctttttttctgcgcgtaatctgctgcttgcaaacaaaaaaaccaccgctaccagcggtggtttgtttgccggatcaagagctaccaactctttttccgaaggtaactggcttcagcagagcgcagataccaaatactgtccttctagtgtagccgtagttaggccaccacttcaagaactctgtagcaccgcctacatacctcgctctgctaatcctgttaccagtggctgctgccagtggcgataagtcgtgtcttaccgggttggactcaagacgatagttaccggataaggcgcagcggtcgggctgaacggggggttcgtgcacacagcccagcttggagcgaacgacctacaccgaactgagatacctacagcgtgagctatgagaaagcgccacgcttcccgaagggagaaaggcggacaggtatccggtaagcggcagggtcggaacaggagagcgcacgagggagcttccagggggaaacgcctggtatctttatagtcctgtcgggtttcgccacctctgacttgagcgtcgatttttgtgatgctcgtcaggggggcggagcctatggaaaaacgccagcaacgcggcctttttacggttcctggccttttgctggccttttgctcacatgttctttcctgcgttatcccctgattctgtggataaccgtattaccgcctttgagtgagctgataccgctcgccgcagccgaacgaccgagcgcagcgagtcagtgagcgaggaagcggaagagcgcctgatgcggtattttctccttacgcatctgtgcggtatttcacaccgcatatggtgcactctcagtacaatctgctctgatgccgcatagttaagccagtatacactccgctatcgctacgtgactgggtcatggctgcgccccgacacccgccaacacccgctgacgcgccctgacgggcttgtctgctcccggcatccgcttacagacaagctgtgaccgtctccgggagctgcatgtgtcagaggttttcaccgtcatcaccgaaacgcgcgaggcagggtgccttgatgtgggcgccggcggtcgagtggcgacggcgcggcttgtccgcgccctggtagattgcctggccgtaggccagccatttttgagcggccagcggccgcgataggccgacgcgaagcggcggggcgtagggagcgcagcgaccgaagggtaggcgctttttgcagctcttcggctgtgcgctggccagacagttatgcacaggccaggcgggttttaagagttttaataagttttaaagagttttaggcggaaaaatcgccttttttctcttttatatcagtcacttacatgtgtgaccggttcccaatgtacggctttgggttcccaatgtacgggttccggttcccaatgtacggctttgggttcccaatgtacgtgctatccacaggaaagagaccttttcgacctttttcccctgctagggcaatttgccctagcatctgctccgtacattaggaaccggcggatgcttcgccctcgatcaggttgcggtagcgcatgactaggatcgggccagcctgccccgcctcctccttcaaatcgtactccggcaggtcatttgacccgatcagcttgcgcacggtgaaacagaacttcttgaactctccggcgctgccactgcgttcgtagatcgtcttgaacaaccatctggcttctgccttgcctgcggcgcggcgtgccaggcggtagagaaaacggccgatgccgggatcgatcaaaaagtaatcggggtgaaccgtcagcacgtccgggttcttgccttctgtgatctcgcggtacatccaatcagctagctcgatctcgatgtactccggccgcccggtttcgctctttacgatcttgtagcggctaatcaaggcttcaccctcggataccgtcaccaggcggccgttcttggccttcttcgtacgctgcatggcaacgtgcgtggtgtttaaccgaatgcaggtttctaccaggtcgtctttctgctttccgccatcggctcgccggcagaacttgagtacgtccgcaacgtgtggacggaacacgcggccgggcttgtctcccttcccttcccggtatcggttcatggattcggttagatgggaaaccgccatcagtaccaggtcgtaatcccacacactggccatgccggccggccctgcggaaacctctacgtgcccgtctggaagctcgtagcggatcacctcgccagctcgtcggtcacgcttcgacagacggaaaacggccacgtccatgatgctgcgactatcgcgggtgcccacgtcatagagcatcggaacgaaaaaatctggttgctcgtcgcccttgggcggcttcctaatcgacggcgcaccggctgccggcggttgccgggattctttgcggattcgatcagcggccgcttgccacgattcaccggggcgtgcttctgcctcgatgcgttgccgctgggcggcctgcgcggccttcaacttctccaccaggtcatcacccagcgccgcgccgatttgtaccgggccggatggtttgcgaccgtcacgccgattcctcgggcttgggggttccagtgccattgcagggccggcagacaacccagccgcttacgcctggccaaccgcccgttcctccacacatggggcattccacggcgtcggtgcctggttgttcttgattttccatgccgcctcctttagccgctaaaattcatctactcatttattcatttgctcatttactctggtagctgcgcgatgtattcagatagcagctcggtaatggtcttgccttggcgtaccgcgtacatcttcagcttggtgtgatcctccgccggcaactgaaagttgacccgcttcatggctggcgtgtctgccaggctggccaacgttgcagccttgctgctgcgtgcgctcggacggccggcacttagcgtgtttgtgcttttgctcattttctctttacctcattaactcaaatgagttttgatttaatttcagcggccagcgcctggacctcgcgggcagcgtcgccctcgggttctgattcaagaacggttgtgccggcggcggcagtgcctgggtagctcacgcgctgcgtgatacgggactcaagaatgggcagctcgtacccggccagcgcctcggcaacctcaccgccgatgcgcgtgcctttgatcgcccgcgacacgacaaaggccgcttgtagccttccatccgtgacctcaatgcgctgcttaaccagctccaccaggtcggcggtggcccatatgtcgtaagggcttggctgcaccggaatcagcacgaagtcggctgccttgatcgcggacacagccaagtccgccgcctggggcgctccgtcgatcactacgaagtcgcgccggccgatggccttcacgtcgcggtcaatcgtcgggcggtcgatgccgacaacggttagcggttgatcttcccgcacggccgcccaatcgcgggcactgccctggggatcggaatcgactaacagaacatcggccccggcgagttgcagggcgcgggctagatgggttgcgatggtcgtcttgcctgacccgcctttctggttaagtacagcgataaccttcatgcgttccccttgcgtatttgtttatttactcatcgcatcatatacgcagcgaccgcatgacgcaagctgttttactcaaatacacatcacctttttagacggcggcgctcggtttcttcagcggccaagctggccggccaggccgccagcttggcatcagacaaaccggccaggatttcatgcagccgcacggttgagacgtgcgcgggcggctcgaacacgtacccggccgcgatcatctccgcctcgatctcttcggtaatgaaaaacggttcgtcctggccgtcctggtgcggtttcatgcttgttcctcttggcgttcattctcggcggccgccagggcgtcggcctcggtcaatgcgtcctcacggaaggcaccgcgccgcctggcctcggtgggcgtcacttcctcgctgcgctcaagtgcgcggtacagggtcgagcgatgcacgccaagcagtgcagccgcctctttcacggtgcggccttcctggtcgatcagctcgcgggcgtgcgcgatctgtgccggggtgagggtagggcgggggccaaacttcacgcctcgggccttggcggcctcgcgcccgctccgggtgcggtcgatgattagggaacgctcgaactcggcaatgccggcgaacacggtcaacaccatgcggccggccggcgtggtggtgtcggcccacggctctgccaggctacgcaggcccgcgccggcctcctggatgcgctcggcaatgtccagtaggtcgcgggtgctgcgggccaggcggtctagcctggtcactgtcacaacgtcgccagggcgtaggtggtcaagcatcctggccagctccgggcggtcgcgcctggtgccggtgatcttctcggaaaacagcttggtgcagccggccgcgtgcagttcggcccgttggttggtcaagtcctggtcgtcggtgctgacgcgggcatagcccagcaggccagcggcggcgctcttgttcatggcgtaatgtctccggttctagtcgcaagtattctactttatgcgactaaaacacgcgacaagaaaacgccaggaaaagggcagggcggcagcctgtcgcgtaacttaggacttgtgcgacatgtcgttttcagaagacggctgcactgaacgtcagaagccgactgcactatagcagcggaggggttggatcaaagtactttgatcccgaggggaaccctgtggttggcatg* | *AtU6-*WT crRNA scaffold-HDV ribozyme-Ploy T-*CaMV 35S promoter-ISAam1-3 X SV40 NLS-CaMV 3'UTR* |

**Supplementary table 2. Amino acid sequence of ISAam1.**

| ISAam1 | MVNKSYKFRLYPTKEQEQLLAKTFGCVRFVYNKMLEERIQIYEKFKDDKEALKKQTFPTPAKYKKEFPWLKEVDSLALANAQLNLQKAFQNFFSGRAGFPKFKNRKAKQSYTTNVVNGNIQLSDGYIKLPKLKWVKFKQHREIPAHHIIKACTIKKTKTGKYYVSILTEYEHQPVPKEIQTVVGLDFSMNGLFVDSEGKRANYPRFYRQALEKLAKEQRILSRRKKGSNRWHKQRLKVAKLHEKIANQRKDFLHKKSYELAKQYDCVVIENLNVKGMSQVLNFGKSVHDNGWGMFRTFLQYKLEEQGKKLIKIDKWFPSSKTCSCCDQVKESLSLSERTFRCECGFESDRDVNAAINIKHEGMKRLAIA* |
| --- | --- |

**Supplementary table 3. Primer sets used in this study.**

| **Name** | **Sequence 5'-3'** | **Description** |
| --- | --- | --- |
| Hi-Aam1-T1F | gagtacggtgtgcAGGTGTTGTCCGAAGCAATGGGG | Primer sets used for amplify ISAam1-T1. |
| Hi-Aam1-T1R | ggatgctggatggAGGTTGAGGGCATTTGGGGTAGT |  |
| Hi-Aam1-T2F | gagtacggtgtgcGATGAAGTGTTTGTCCCAAAACC | Primer sets used for amplify ISAam1-T2. |
| Hi-Aam1-T2R | ggatgctggatggGAGTGTGACGAGAAGAGAAACAG |  |
| Hi-Aam1-T34F | gagtacggtgtgcGCAACAGCACGTGGAGCTAAAAC | Primer sets used for amplify ISAam1-T3 and ISAam1-T4. |
| Hi-Aam1-T34R | ggatgctggatggAGACGATTCCCGAAGAAGACTAA |  |
| Hi-Aam1-T56F | gagtacggtgtgcGATGAAGCACTGGCAGACCTGGT | Primers to amplify ISAam1-T5 and ISAam1-T6. |
| Hi-Aam1-T56R | ggatgctggatggCAGGAGCATTTTGCTTTGCGTCC |  |
| Hi-Aam1-T78F | gagtacggtgtgcTCATATACACATCACTATTGGTG | Primer sets used for amplify ISAam1-T7 and ISAam1-T8. |
| Hi-Aam1-T78R | ggatgctggatggCCAGCCCCACGGTTCATCAGATA |  |
| Hi-Aam1-T9F | gagtacggtgtgcGGAAGTGGATGATGACAAAG | Primer sets used for amplify ISAam1-T9. |
| Hi-Aam1-T9R | ggatgctggatggCAGCAAGCAACAAACATAGA |  |
| Hi-GmPDS-F | ggagtgagtacggtgtgcGGATTGGCTGGTTTATCAACTGC | Primer sets used for amplify GmPDS-T1 and GmPDS-T2. |
| Hi-GmPDS-R | gagttggatgctggatggCCTGATGATGAAGTTATAAAGCC |  |
| Hi-GmWRKY28-F | ggagtgagtacggtgtgcCAGTGAAACCCTTGCGACCC | Primer sets used for amplify GmWRKY28-T1 and GmWRKY28-T2. |
| Hi-GmWRKY28-R | ggagtgagtacggtgtgcGGGAACCAAAAGCCAGCAG |  |
| Hi-GmCHR6-F | ggagtgagtacggtgtgcTGACGTGAAGGGTGTGTGGG | Primer sets used for amplify GmCHR6-T1. |
| Hi-GmCHR6-R | ggagtgagtacggtgtgcTATCTCATCCACTGGCCC |  |
| Hi-GmSCL1-F | ggagtgagtacggtgtgcGAAATTTTGTATGAGCTGAGT | Primer sets used for amplify GmSCL1-T1 and GmSCL1-T2. |
| Hi-GmSCL1-R | ggagtgagtacggtgtgcAAGTCATCTTAGAAGGTTC |  |
